# Beta cyclodextrin stabilized cupric oxide nanoparticles assisted thermal therapy for lung tumor and its effective in vitro anticancer activity

**DOI:** 10.1038/s41598-025-96578-3

**Published:** 2025-08-07

**Authors:** Anakha D. Rajeeve, Vyshnavi T. Veetil, Sabarinathan Palaniyappan, Ramasamy Yamuna, Vishal Bhalla

**Affiliations:** 1 Department of Chemistry, Amrita School of Physical Sciences Coimbatore, Amrita Vishwa Vidyapeetham, Coimbatore, India; 2 Bio-Materials Chemistry Research Laboratory, Amrita School of Engineering Coimbatore, Amrita Vishwa Vidyapeetham, Coimbatore, India; 3 Center for Biosystems and Machines, King Fahd University of Petroleum and Minerals, Dhahran, Saudi Arabia; 4 School of Energy and Environment, NICMAR University, Pune, Maharashtra India

**Keywords:** CuONPs@βCD, Temperature distribution, Anticancer, Lung cancer, Antimicrobial, COMSOL multiphysics, Cancer, Computational biology and bioinformatics, Drug discovery

## Abstract

The unique physicochemical properties of cupric oxide nanoparticles (CuO NPs) make them suitable for a wide range of therapeutic applications. Here, we synthesized β-cyclodextrin (βCD) capped CuO NPs (CuONPs@βCD) using a simple reduction process. The formation and physicochemical characteristics were identified via different spectroscopic techniques. The CuONPs@βCD displayed antimicrobial activity as good as commercial drugs. Dimethyl thiazolyl tetrazolium bromide (MTT) assay was carried out to assess the anticancer properties of CuONPs@βCD against A549 lung cancer cells. The result demonstrated that the anticancer activity of CuONPs@βCD with IC_50_ values of 41.06 ± 0.05 and 19.46 µg/mL at 24 and 48-h incubation period, respectively. CuONPs@βCD exhibited anticancer activity on A549 lung cancer cells while having less adverse effects on normal cells. Annexin V-FITC/PI assay, reactive oxygen species (ROS) analysis, disruption of mitochondrial membrane potential (Δψm), and AO/EB apoptosis studies in A549 cells revealed significant apoptotic impact of CuONPs@βCD when compared to the control. Moreover, thermal therapy study of CuONPs@βCD in lung tumor using COMSOL Multiphysics has been reported. Our investigation revealed Case III, where the temperature distribution at the top surface of the tumor is best and may be the most effective way to treat lung cancer. It was found that an incident flux of 8000 Wm^− 2^ for 900 s and an extinction coefficient of 8.266 m^− 1^ for CuONPs@βCD were the best conditions for reaching a temperature of 43.63 °C across the whole tumor area. Thus, these findings open new research opportunities and potential use of CuONPs@βCD for biological applications.

**Supplementary Information:**

The online version contains supplementary material available at 10.1038/s41598-025-96578-3.

## Introduction

Lung cancer continues to be a major cause of death worldwide. Chemotherapeutic chemicals such as alkylating metabolites have low specificity for targeting cancer cells which may destroy healthy cells too^1,2^. Consequently, there is an urgent need for inventive therapeutic approaches that can offer more precise and efficient therapy alternatives. Thermal therapy is a promising method that uses heat to destroy malignant tissues^3^. This approach has attracted considerable attention because of its minimally invasive characteristics and potential for precise localization. Nanotechnology has become a crucial factor in improving the effectiveness of thermal therapy. Hyperthermia therapy involving nanoparticles (NPs), and ionizing radiation is efficient in increasing the radiosensitivity of tumor cells^4–7^. This treatment paves the way for tailoring radiation dosage and duration of hyperthermia, and therefore, maximizing the sensitization and vulnerability of cancer cells to radiation due to its poor defense mechanism while limiting the death of surrounding tissue cells. Numerous studies have shown that cupric oxide (CuO) NPs can specifically cause DNA damage and cytotoxicity in the A549 human lung cancer and chronic myelogenous leukemia K562 cell lines^8,9^. CuO NPs have gained significant interest among other metal-based nanomaterials due to their distinct physicochemical characteristics, such as exceptional thermal conductivity, biocompatibility, and the capacity to produce localized hyperthermia in response to certain stimuli^10,11^. CuO NPs can be designed to specifically aggregate in tumor tissues, thus optimizing the therapeutic impact while reducing harm to adjacent healthy tissues. The report by Hildebrandt et al. suggests the cellular and molecular basis of hyperthermia^12^. Additionally, Bhoi and Esmaeili et al. studies imply the magnetic hyperthermia and anticancer effects of CuO NPs^13,14^. Moreover, copper is the vital metal for respiration process, regulation of neurotransmitter, synthesis of collagen proteins, metabolism of key elements such as iron, crucial component of many enzymes and proteins^15^. Therefore, prospective use of CuO as anticancer drugs is indispensable in the treatment of kidney, prostate, lung, breast, and glioma.

Thermal therapy, encompassing hyperthermia, is a treatment method that employs heat to eradicate cancer cells or enhance their susceptibility to cancer therapies, including radiation and chemotherapy^16^. However, possible side effects encompass local reactions such as pain, burns, swelling, and bleeding, in addition to systemic reactions including nausea, vomiting, and fatigue.

Recent literature studies demonstrate that lung cancer patients are vulnerable to microbes such as virus, bacteria and fungus^17^. CuO NPs have tremendous biomedical applications such as antifungal and antibacterial properties^18^. These characteristics have led to the creation of several strategies that have direct applications in the biomedical industry. However, there remain several challenges and gaps in current research, including the need for improved targeting specificity, reduced toxicity to healthy cells, and enhanced understanding of the interaction mechanisms at the molecular level. However, excessive copper intake may lead to hemolysis, jaundice, and potentially fatal outcomes^19^. The toxicity of CuO is contingent upon the level of exposure and individual characteristics. Size, shape and morphology of CuO NPs are crucial for their antimicrobial and anticancer activities^20,21^. Therefore, it is necessary to choose suitable non-toxic capping agents to reduce the side effects of hyperthermia involving CuO NPs and improve their anticancer activities.

Cyclodextrins (CDs), derived from the starch enzymatic degradation, are well-known toroidal-shaped oligosaccharides comprising six to eight glucose units connected through α-1,4-glycosidic bonds. Among the CDs, βCDs are mainly utilized as a capping agent for the synthesis of metal oxide NPs owing to their availability, low cost, moderate ring size, and appropriate hydrophilic outer surface which stabilizes the metal oxide NPs^22^. The biocompatibility of βCD and its capacity to enable controlled release of therapeutic agents makes it a significant asset in the advancement of nanomedicine platforms. Thus, interaction among the unique auxiliary properties of cyclodextrins and the extraordinary properties of copper-based materials can potentially enhance the physiochemical characteristics of CuO NPs^20,23,24^.

Therefore, we investigated the lead of β-CD capped CuO NPs (CuONPs@βCD) to explore the biological applications that have not been previously reported. Here, the antibacterial and antifungal activities of CuONPs@βCD are reported. Dimethyl thiazolyl tetrazolium bromide (MTT) assay and apoptosis have been carried out to assess the anticancer properties of CuONPs@βCD against A549 lung cancer cells. Furthermore, we explored the application of CuONPs@βCD as thermal therapeutic agent for the treatment of lung tumor using COMSOL Multiphysics software. We used an average temperature of 37 °C as the initial condition to analyze the spatiotemporal temperature field, as both the body and the lung typically maintain a temperature of about 37 °C^25^. Consequently, the objective of this study is to make a substantial impact on the temperature distribution within a lung tumor that is embedded with CuONPs@βCD and surrounded by healthy tissue. Moreover, we reported anticancer and antimicrobial activities of CuONPs@βCD which further support the thermal therapy towards lung cancer treatment. Through this investigation, we aim to prove that CuONPs@βCD are effective agents for thermal therapy, providing a novel and promising approach to the treatment of lung cancer.

## Materials and methods

### Materials

For the synthesis of CuONPs@βCD: β-CD, Cu(NO_3_)_2_·3H_2_O, and NaBH_4_ were procured from Molychem, NICE chemicals, and Sigma-Aldrich, respectively.

### Characterizations

The chemical structure of CuONPs@βCD was analyzed using FT-IR spectroscopy [Bruker (Alpha Platinum)] within the range of 4000–400 cm^−1^. The purity and structure of CuONPs@βCD were evaluated using XRD patterns obtained from a Malvern Panalytical multipurpose model, employing Cu Kα radiation within the 2θ range of 10−70° and a step size of 0.02°. The high-resolution transmission electron microscopy (HR-TEM) images were captured using a 200 kV TEM (Tecnai G2 F20 X-TWIN). The particle size and surface charge of CuONPs@βCD were determined using the Zetasizer Nano-ZS (Malvern Panalytical) instrument.

### Synthesis of CuONPs@βCD

CuONPs@βCD were synthesized using a simple chemical reduction procedure as reported recently from our lab^23^. In general, β-CD (0.093 g, 0.082 mmol) and Cu(NO_3_)_2_·3H_2_O (0.077 g, 0.318 mmol) were mixed with 10 mL of milli-Q water in a round bottom flask. After being subjected to sonication for several minutes, this solution mixture was stirred vigorously on a magnetic stirrer for 1 h. Subsequently, 0.5 mL of freshly made NaBH_4_ (1.9 mmol in 2 mL of water) was added dropwise and stirred constantly for 2 h. We detected brownish black CuO NPs upon reduction and recovered them via centrifugation at 1500 rpm. Finally, the solid residue was washed and subsequently dried at 80 °C under vacuum conditions.

### Antibacterial activity

The antibacterial activities of CuONPs@βCD were determined using the disc diffusion method on nutrient agar. *Staphylococcus aureus* (*S. aureus*, (G+)) and *Escherichia coli* (*E. coli*, (G–)) were utilized as bacterial strains and grown on agar nutrient. Under specific conditions, nutrient agar plates were inoculated with a bacterial strain and spread using a glass L-rod containing 100 µL of the grown culture. Subsequently, different concentration of CuONPs@βCD (25, 50, 75 and 100 µL) and the control (chloramphenicol, 100 µL) were placed on agar plates and incubated for a full day at 37 °C. The zone of inhibition (ZOI) was calculated and reported in millimeters (mm) following incubation^26^.

### Antifungal activity

The antifungal activities of CuONPs@βCD were evaluated via the disc diffusion technique against *Candida albicans* (*C. albicans*), which was cultured in yeast extract peptone dextrose (YEPD) broth. Autoclaved YEPD broth agar was plated and allowed to cool. Using an L-rod, cultured *C. albicans* were spread-plated onto YEPD broth agar plates once the agar solidified. The agar plate was perforated with holes by using a sterile well cutter. On the punched-in wells, different concentrations of CuONPs@βCD (25, 50, 75 and 100 µL) and the control drug (fluconazole, 100 µL) were added to each plate and incubated at 37 °C for 24 h. The zone of inhibition was computed and reported in millimeters (mm) following incubation^26,27^.

### In vitro anticancer activities

#### Cell culture

The A549 human lung cancer and HEK293 (normal cell, embryonic kidney-derived) cell lines were acquired from the American Type Culture Collection (ATCC). It was developed in Dulbecco’s modified Eagle’s medium (DMEM) supplemented with Fetal Bovine Serum (FBS, 10%) and penicillin-streptomycin (1%) in a humidified atmosphere of 5% CO_2_ at 37 °C.

#### Cytotoxicity assay

The cytotoxic effect of synthesized CuONPs@βCD was analyzed using HEK293 (normal cell, embryonic kidney-derived) and A549 (lung cancer) cell lines using dimethyl thiazolyl tetrazolium bromide (MTT) assay. About 100 µL of the diluted cell suspension (50,000 cells per well) was seeded in each 96-well microtiter plate and incubated for 24 h. The synthesized CuONPs@βCD and cisplatin (standard drug as control) solution at concentrations ranging from 0 to 100 µg/mL was added to the partial monolayer and incubated up to 24 h and 48 h. The plates were incubated in an environment with 5% CO_2_ for 24 h at 37 °C. After the incubation period, 100 µL MTT was added to each well and further incubated for 4 h at 37 °C in a 5% CO_2_ atmosphere. After removing the supernatant, 100 µL of dimethyl sulfoxide (DMSO) was added to solubilize the ensuing formazan product produced by mitochondrial reduction of MTT. At a wavelength of 590 nm, the absorbance was measured using a microplate reader^28^.

#### Selectivity index

The selectivity index (SI) was computed using the following formula to quantify the degree of selectivity to ascertain the anticancer potential of CuONPs@βCD and cisplatin:

1 $$\:SI=\:\frac{{IC}_{50}\:normal\:cell\:line}{{IC}_{50}\:cancer\:cell\:line}$$

#### Detection of reactive oxygen species using carboxy-H_2_DCFDA

A fresh stock solution of carboxy-H_2_DCFDA in sterile DMSO was prepared. Following a 24 h treatment with synthesized CuONPs@βCD at the IC_50_ concentration, carboxy-H_2_DCFDA was employed at a final concentration of 1 µM in a standard culture medium with reduced serum (2%). The cultures were incubated in a conventional incubator for 30 min in the dark at 37 °C with 5% CO_2_. All unused dye solutions were discarded. Carboxy-H_2_DCFDA containing medium was removed, and the samples were washed twice with PBS. Furthermore, A549 lung cancer cells were protected from light. These cell cultures were incubated after adding CuONPs@βCD to the carboxy-H_2_DCFDA-loaded A549 cells as desired. Immediately, the A549 cells were observed with a fluorescent microscope (Olympus, CKX-53, Japan), and the percentage of dead A549 cells was calculated in at least three random microscopic areas^29^.

#### Assessment of mitochondrial membrane potential (∆ψm)

The Δψm was assessed using 5,5′,6,6′-tetrachloro-1,1′,3,3′-tetraethylbenzimidazolcarbocyanine iodide (JC-1) cationic fluorescent dye. In brief, 5 × 10^5^ cells/well were seeded in 6-well plates and incubated overnight at 37 °C with 5% CO_2_ for attachment. After overnight attachment, the cells were treated with fresh medium containing CuONPs@βCD (41.06 µg/mL). After incubation for 12 and 24 h, a working stock of 1–10 M JC-1 in cell culture medium was prepared. To prepare a 1 µM working stock, 5 µL of JC-1 (200 µM) was incorporated into 1 mL of cell culture medium. Further, the plates were incubated at 5% CO_2_ and 37 °C for a duration of 15 to 30 min. Immediately, the cells were visualized using a fluorescence microscope (Olympus, CKX-53, Japan), and the percentage of dead cells was quantified in at least three random microscopic fields^30^.

#### Acridine orange/ethidium bromide staining

The assessment of cell apoptosis in response to CuONPs@βCD and cisplatin sample treatment was conducted using acridine orange (AO) and ethidium bromide (EB) fluorescence labelling. Briefly, 4000 cells/well were seeded into 24-well plates and incubated for 24 h at 37 °C. Thereafter, CuONPs@βCD and cisplatin samples were added to the cells and incubated for 24 h. Each well (500 µL) was added with 10 µL of the staining solution containing AO (100 µg/mL) and EB (100 µg/mL). After a 24 h incubation period, the cells were observed immediately using a fluorescence microscope (Olympus, CKX-53, Japan), and the proportion of dead cells was measured in at least three randomly selected microscopic areas^31^.

#### Annexin V-FITC/PI staining by flow cytometry

A549 cells were seeded in a 6-well plate (10^5^ cells/well) and cultured at 37 °C for 24 h. Next, the cells were incubated with IC_50_ concentration of CuONPs@βCD (41.06 µg/mL) for 24 h. Then, the cells were trypsinized and washed with PBS. Next, the cells were stained with annexin V-FITC/PI according to the annexin V-FITC apoptosis detection kit. Finally, apoptosis was evaluated by a flow cytometer (SYSMEX, Japan), and the data were analyzed by Flow Jo software. The cells without any treatment were used as blank control^32^.

### Statistical analysis

Statistical analysis was performed using an unpaired t-test for in vitro experiment to compare the cytotoxicity of all the test samples. All biological experiments were conducted at least in triplicate, and the results are expressed as mean ± standard deviation (SD). Graph Pad Prism software (10.1.0) was utilized to perform statistical analysis.

## Theoretical modeling on the temperature distribution within a lung tumor

**Fig. 1:**
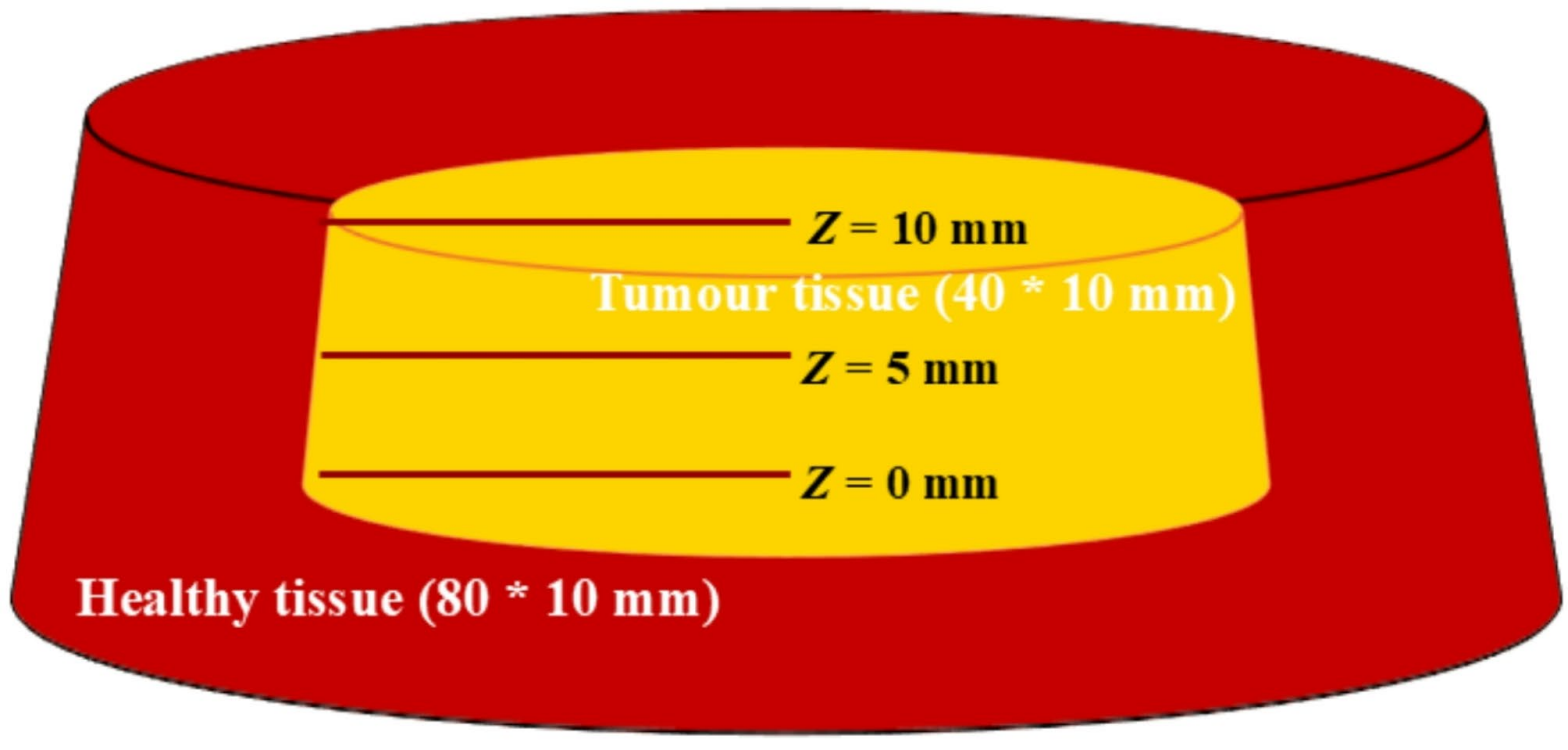
Schematic illustration of the tumor region surrounded by healthy lung tissue. Three cases of spatial distribution of temperature along the depths (*Z* = 0, 5, and 10 mm) of tumor.

The 3D hyperthermia process was demonstrated with the assumption that a distinctive lung cancerous tumor comprises a cylindrical coordinate of 40 mm in diameter and 10 mm in depth, surrounded by healthy tissue with a diameter of 80 mm and a depth of 10 mm, as displayed in Fig. 1. Cylindrical lung tumor was considered in earlier investigations^33,34^. Since tumors exhibit irregularity with small, oval-shaped cells, a cylindrical form can effectively represent the overall structure of specific tumor forms, particularly in molecular modeling contexts. The cylindrical geometry facilitates the simplification of equations related to heat transfer and temperature distribution, rendering them suitable for analytical or numerical approaches^35^. Small-cell lung cancer generally arises in central airways; thus, a cylindrical model aligned with airway structures can effectively approximate the tumor’s geometry in relation to the airways, aiding in the representation of airflow and temperature variations surrounding the tumor^36^. It was assumed that the top surface of the lung tumor was subjected to irradiation. COMSOL Multiphysics was used to design and accomplish 3D numerical models based on applying the bioheat transfer time-dependent method. This study examined the temperature distribution in a lung tumor embedded with CuONPs@βCD and surrounded by healthy tissue in three distinct ways. Figure 1 provides visual representations of these.

Case I: The distribution of temperature within the tumor surface embedded with CuONPs@βCD is also illustrated in Fig. 1 and is denoted to as Case I in this manuscript. The tumor distributes the temperature at the bottom of 0 mm-depth (*Z*).

Case II: The illustration in Fig. 1 also suggests the temperature distribution in the middle of the tumor area, with a radius of 20 mm and a depth of 5 mm. The article refers to this as Case II.

Case III: The third case was chosen due to the temperature distribution at the top surface of the tumor, with a radius of 20 mm and depth of 10 mm. Figure 1 also illustrates this case, which was considered the baseline for our investigation. Throughout this paper, this distribution is referred to as Case III.

### Thermo-physical properties of CuONPs@βCD

In this study, we introduced CuONPs@βCD in lung tumor tissue to enable localized heating via photothermal treatment. CuONPs@βCD exhibit extinction properties by absorbing and scattering light at the irradiated wavelength. The extinction coefficient, which incorporates the effects of absorption and scattering, has been assumed to be *k*_*e*_^37^.

In the modelling it has been assumed that the amount of the nanoparticles is very less (|*m*|<<1, m is complex refractive index [*m* = *n* + i*κ*]) and they are following the Rayleigh scattering. The emission from the nanoparticles has been neglected because the temperature rise is not more than 41 °C. Thus, the rate of change of the irradiation with respect to the depth has been calculated by using Beer Lambert’s law, Eq. (2).

2 $$\:I={I}_{o}\text{exp}\left(-{k}_{e}*y\right)$$

where, *I*_o_ is the incident intensity, $\:{k}_{e}$ denotes the extinction coefficient and $\:y$ represents the depth of the tumor.

We explain how to use Beer-Lambert’s law in terms of light intensity and demonstrate its application in our COMSOL simulations to provide a detailed and understandable description of our modeling approach.

### Thermo-physical properties of healthy and the tumor lung tissue

Thermo-physical properties refer to the measurable characteristics that determine the structure, function, and behavior of both healthy and tumor lung tissue. Comprehending these qualities is essential to understand the normal functioning of tissues and the alterations they undergo in the presence of disease. These features have an impact on how lung tissues react to physiological processes, medicinal therapies, and diagnostic imaging. Modifications to the physical properties of tumor tissues can influence their detection and the efficacy of their medical treatment. Tables S1 and S2 contain the combined values of physical attributes for both healthy tissue and tumor.

### Biological heat transfer technology

#### Tissue heat transfer equation

Biological heat transfer models compute the heat transfer within biological tissue. Generally, the temperature closest to the emission slot is the highest. The heat source’s effect diminishes with distance, and the rate at which the temperature rises slows down. The Pennes equation is the most commonly utilized lung tissue model^34,38^. The Pennes equation, a basic bioheat transfer model, describes the temperature distribution in biological tissues. It considers a few variables that affect heat transfer, such as blood perfusion and metabolic heat generation.

Pennes Equation:

3 $$\rho{C}_{p}\frac{\partial T}{\partial t}=\:{k}_{t}*\left({\nabla }^{2}\right.T)+{\rho}_{b}{C}_{b}{\omega }_{b}({T}_{b}-T)+{Q}_{m}$$

where *ρ* is the density of lung tissue (kg/m^3^) and *ρ*_b_ is the density of blood (kg/m^3^). *C*_p_ is the specific heat capacity of lung tissue (J/kg K), while *C*_b_​ is the specific heat capacity of blood (J/kg K). *T* is the temperature of lung tissue in C, while *T*_b_ is the temperature of blood in C. Thermal conductivity (W/mK) is denoted by *k*_t_. Blood perfusion rate (s^− 1^) is designated by *ω*_b_. *Q*_m_ is the metabolic heat source (W/m^3^).

Metabolic heat source: In bioheat transfer simulations, it is a term used to describe the heat produced by biological tissues metabolic processes^39^. A volumetric heat-generating term (*Q*_m_) often represents the metabolic heat source in the bioheat equation. This parameter represents the rate of heat generation per unit volume of tissue. The standard unit of measurement is W/m^3^.

## Results and discussion

CuONPs@βCD were synthesized utilizing a previously reported chemical reduction method from our laboratory^23^. The investigation of metal oxide NPs in cancer treatment is gaining significant attention due to the potential for customizing the physicochemical properties of NPs, thereby enhancing their biocompatibility and interactive capabilities through surface functionalization. The primary objectives of surface functionalization are to enhance biocompatibility, thereby reducing toxicity, promoting the body’s acceptance of NPs and directing NPs specifically to diseased cells or tissues, which maximizes therapeutic efficacy while minimizing side effects^40^.

**Fig. 2:**
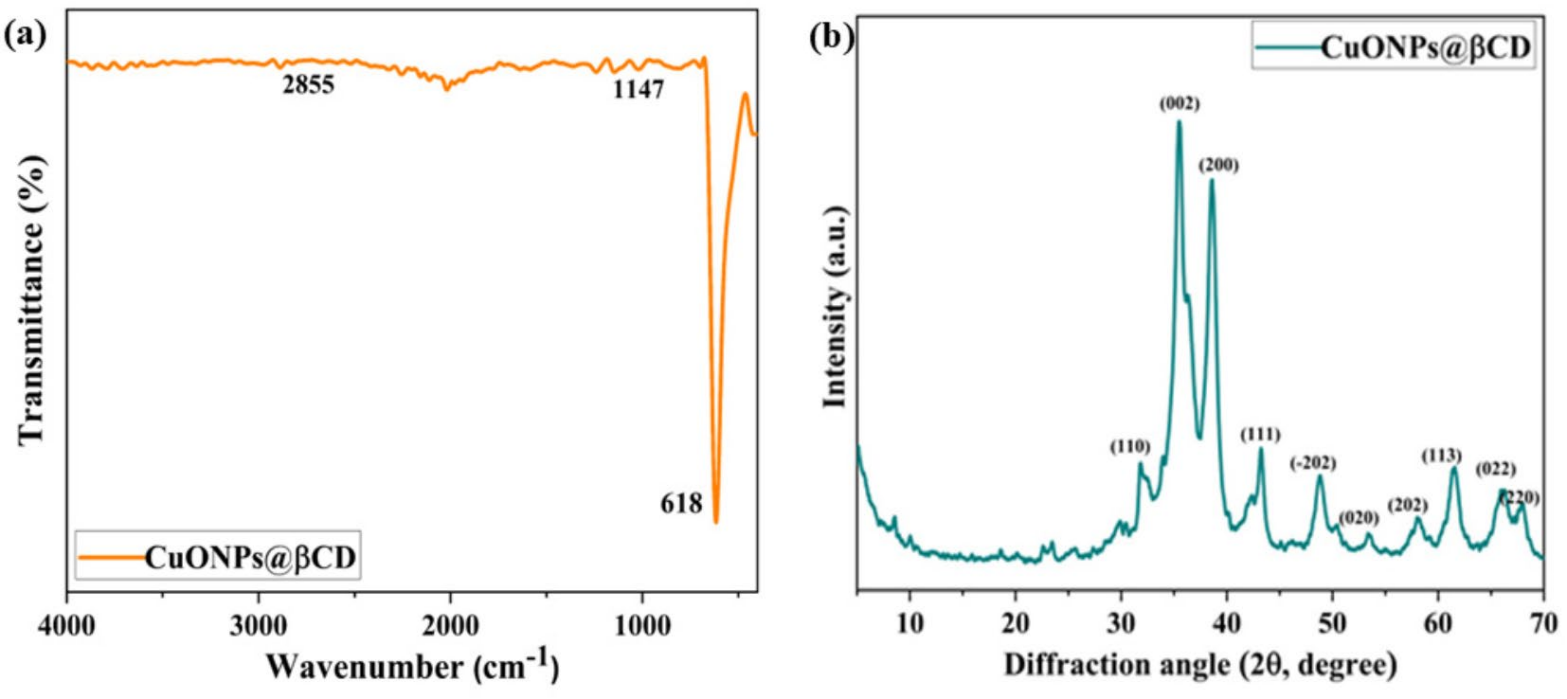
(**a**) FT-IR spectrum and (**b**) XRD pattern of CuONPs@βCD.

The formation of CuONPs@βCD is validated using Fourier transform infrared (FT-IR) and powder X-ray diffraction (XRD) analyses, as seen in the Fig. 2a, b, respectively. The FT-IR spectrum of CuONPs@βCD provided significant information about the successful synthesis and interaction between the components. Characteristic peaks of βCD, such as C–H (2855 cm^− 1^) and C–O–C (1147 cm^− 1^) stretching vibrations of glycosidic linkages, confirmed its presence. CuONPs@βCD exhibited a prominent band around 618 cm^− 1^, which corresponds to Cu(II)–O, confirming the formation of pure CuO NPs on the βCD as depicted in Fig. 2a. Therefore, FT-IR analysis confirmed the formation of CuONPs@βCD and elucidated the interactions among the components and supports in evaluating the purity of the synthesized material.

Furthermore, the purity and structure of CuONPs@βCD was evaluated using XRD patterns. The crystal phase of CuO NPs aligns with ICDD card no. 801,916, as evidenced by all diffraction peaks illustrated in Fig. 2b. The distinct peaks at 32.13, 35.54°, 38.55°, 42.90°, 48.78°, 53.40°, 58.32°, 61.63°, 66.03°, and 68.04° correspond to the miller indices (110), (002), (200), (111), (202), (020), (202), (113), (022), and (220), respectively, indicating the monoclinic structure of copper in CuO NPs^15^. The mean crystallite size of CuO NPs, calculated from the full width at half maximum using Debye Scherrer’s formula, is 9.36 nm.

**Fig. 3:**
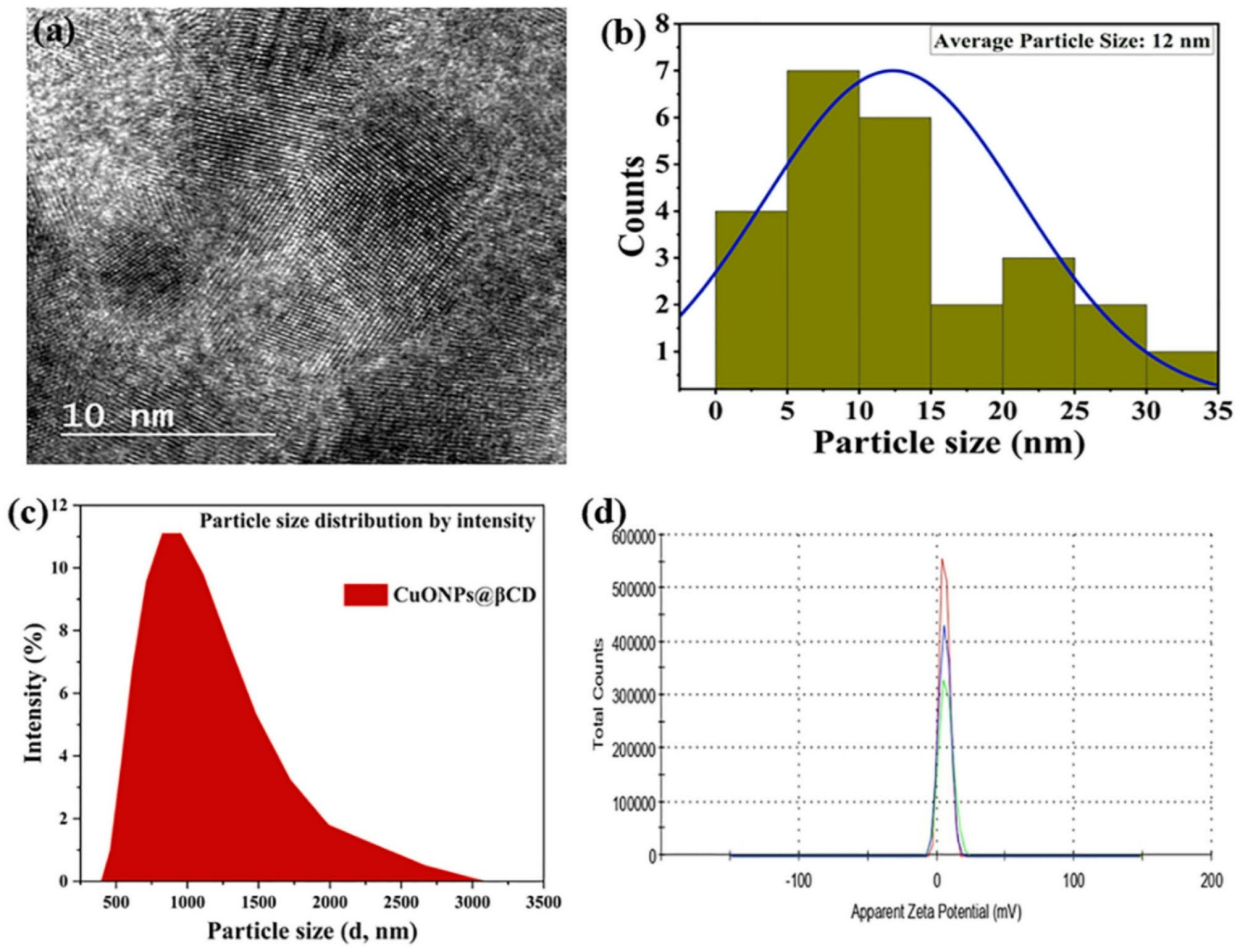
(**a**) HRTEM micrograph, (**b**) particle size distribution histogram obtained from TEM images. DLS analysis of CuONPs@βCD (**c**) particle size distribution (diameter in nm) and (**d**) zeta potential.

HR-TEM image of CuONPs@βCD (Fig. 3a) revealed that the synthesized CuONPs@βCD exhibited a spherical shape on the surface of βCD, characterized by a distinct rough appearance^23^. Figure 3b presented the particle size distribution histogram of synthesized CuO NPs. The mean particle size of CuO NPs is estimated to be 12 nm. Therefore, HR-TEM analysis confirmed the formation of CuONPs@βCD.

Dynamic light scattering (DLS) analysis was carried out to estimate the size and surface charge of the prepared CuONPs@βCD, as shown in Fig. 3c, d. The hydrodynamic size (Z-average diameter) obtained in DLS studies is 895 nm (Fig. 3c) with the polydispersity index (PDI) value of 0.184. The larger particle size of 895 nm can be attributed to water layer covering the surface of NPs due to electrostatic attraction, and it also includes supramolecular capping agent^41,42^. It is generally accepted that particles with even 5 μm diameter are suited to reach and deposit within the lungs^43^. Moreover, a PDI of 0.3 and below is considered to be acceptable with good homogeneity in drug delivery applications^44^. Therefore, literature study reveals that particle size with 895 nm and PDI value of 0.184 is well suited for alveolar intravenous drug delivery^42,44,45^.

Moreover, the stability of material and the surface area charge are both revealed by the zeta potential (ZP) values. Figure 3d presents the average ZP value for CuONPs@βCD in double distilled water, measured at 5.73 mV. ZP exhibiting a more positive or negative charge signifies superior physical colloidal stability, attributed to electrostatic repulsions among particles^46,47^. A positive ZP typically signifies favorable colloidal stability of NPs within a dispersion. A positive ZP minimizes aggregation, thereby ensuring that NPs remain well-dispersed in the solution. This is critical for retaining their desired features and functioning, such as the capacity to interact with biological systems to produce antimicrobial or anticancer effects.

### Biological studies

#### Antibacterial activity

**Fig. 4:**
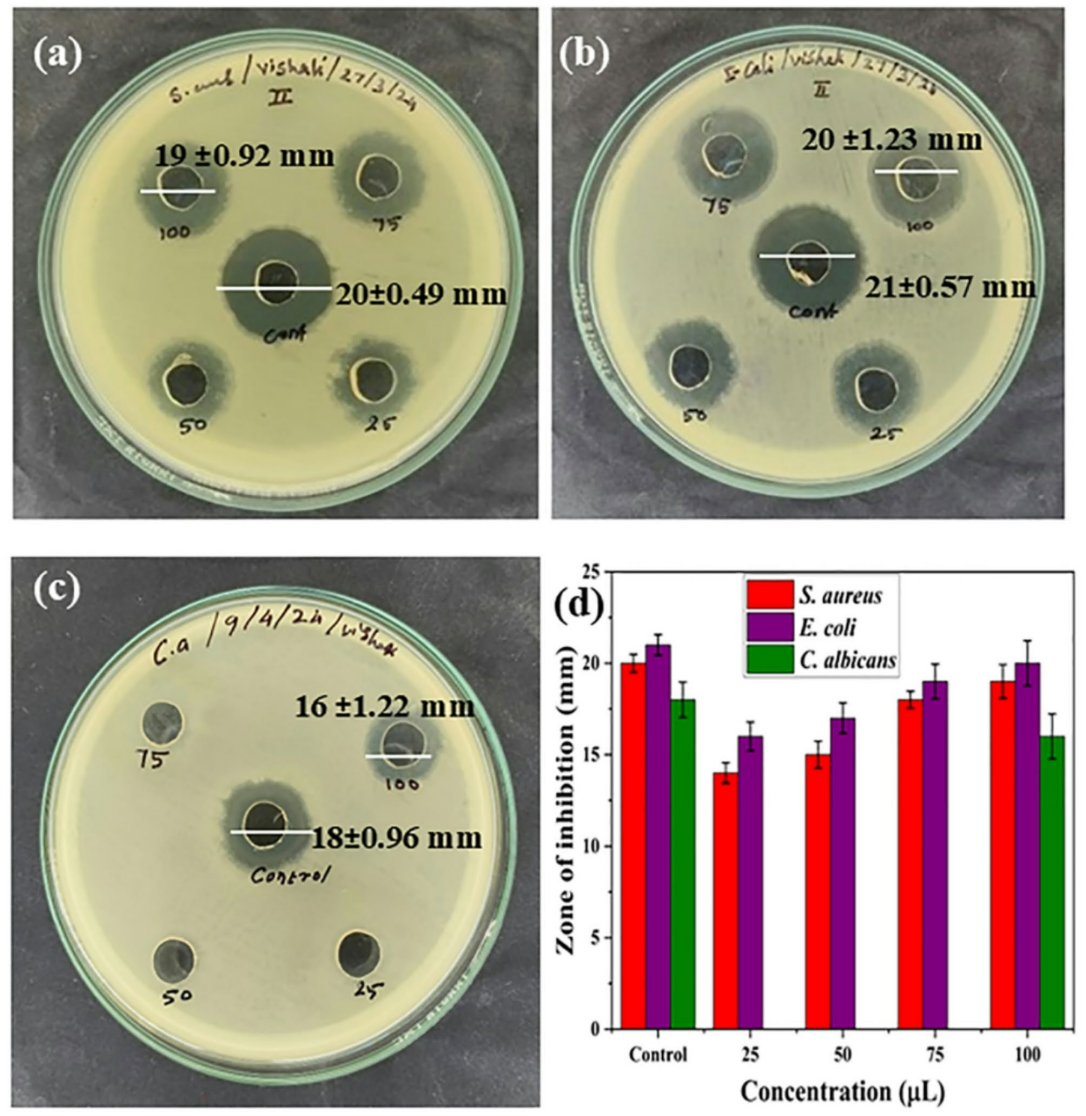
In vitro antibacterial and antifungal activity of CuONPs@βCD tested against (**a**) *S. aureus*; (**b**) *E. coli*; (**c**) *C. albicans*, and (**d**) bar graph representing the ZOI of control (100 µL) & CuONPs@βCD (25, 50, 75, and 100 µL).

The antibacterial activities of synthesized CuONPs@βCD against gram-positive (*S. aureus*) and gram-negative (*E. coli*) bacteria using the disc diffusion technique on nutrient agar are displayed in Fig. 4a, b. Standard antibiotic chloramphenicol has been utilized as a control. Figure 4a, b show that CuONPs@βCD have the highest ZOI against *S. aureus* (19 ± 0.92 mm) and *E. coli* (20 ± 1.23 mm), respectively, at concentrations of 100 µL. Moreover, it is clear from Fig. 4a, b that the ZOI of CuONPs@βCD is close to the ZOI of the control (chloramphenicol). Moreover, the antibacterial activity bar graph representing the ZOI of CuONPs@βCD is depicted in Fig. 4d.

The antibacterial mechanism of CuO NPs is due to the production of reactive oxygen species (ROS), also referred to as free radicals^48,49^. The main sources and controllers of ROS are mitochondria^50^. Thus, excess ROS produced by the NPs may damage mitochondria of bacteria^51^. ROS such as H_2_O_2_, OH•, O_2_•–, etc. will be produced when CuO NPs interact with the bacterial cell wall^24,52,53^.

#### Antifungal activity

Numerous studies have revealed that CuO NPs display antifungal action against a wide variety of fungal infections, including *Candida albicans* (*C. albicans*), *Alternaria alternate* (*A. alternate*), and *Fusarium solani* (*F. solani*)^54,55^. The disc diffusion technique is used to test the antifungal properties of synthesized CuONPs@βCD (Fig. 4c) against *C. albicans* fungal isolates. As shown in Fig. 4c, the fluconazole drug was used as a control, illustrating the establishment of the ZOI against *C. albicans*. Moreover, the antifungal activity bar graph representing the ZOI of CuONPs@βCD is depicted in Fig. 4d. The measured ZOI of the synthesized CuONPs@βCD is approximately 16 ± 1.22 mm against the *C. albicans* fungal strain. Whereas the measured ZOI for control was close to the ZOI of test sample, which indicates the antifungal nature of CuONPs@βCD.

#### Anticancer activity

**Fig. 5:**
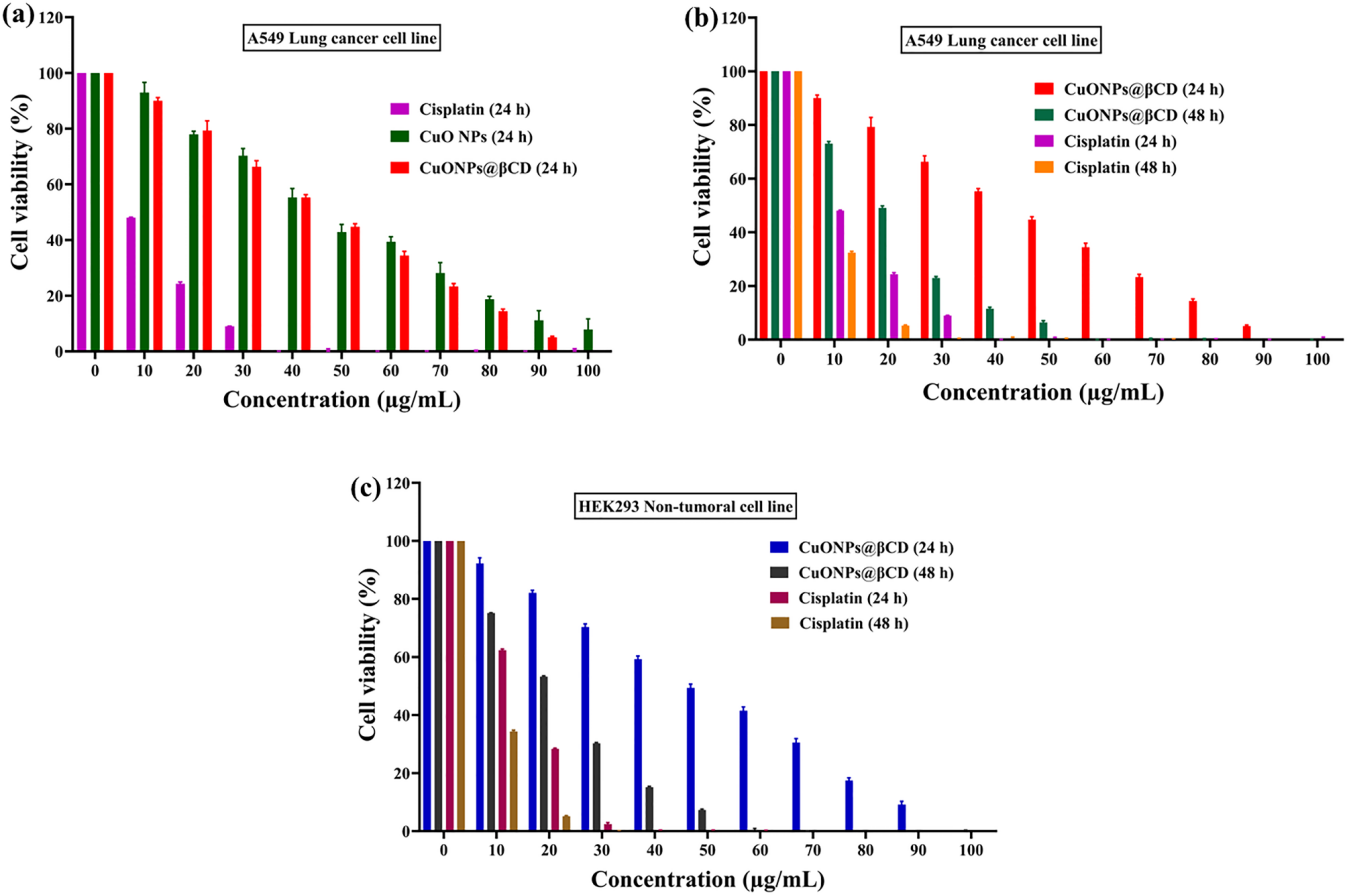
The plot of cell viability in (**a**) Cisplatin, CuO NPs, and CuONPs@βCD (24 h), & (**b**) CuONPs@βCD and cisplatin (24 and 48 h) against A549 in different concentrations, and (**c**) CuONPs@βCD and cisplatin (24 and 48 h) against normal cell (HEK293), the values were expressed as mean ± SD for three independent experiments.

MTT assay was carried out to assess the anticancer efficacy of CuONPs@βCD over 24 and 48 h against A549 human lung cancer cell lines at different concentrations (0–100 µg/mL). The cytotoxic potential of CuONPs@βCD has been compared with that of a conventional drug, cisplatin. Furthermore, the efficacy of CuONPs@βCD is evaluated by comparing it to a negative control CuO NPs, which is chemically synthesized using ascorbic acid as a reducing agent. The percentage of cell viability against varying concentrations of cisplatin, CuO NPs, and CuONPs@βCD during the 24-h incubation period is illustrated in Fig. 5a. CuO NPs, CuONPs@βCD, and cisplatin (24 h incubation period) at 10 µg/mL demonstrated 92.99% ± 3.63, 90.01% ± 1.15, and 48.05% ± 0.15 cell viability; while at 50 µg/mL, cell viability dropped to 42.84% ± 2.73, 44.74% ± 1.09, and 0.46% ± 0.43, and further decreased almost up to 7.86% ± 3.78, 0% ± 0.04, and 0% ± 0.44 at 100 µg/mL, respectively. In addition, the percentage cell viability against varying concentrations of CuONPs@βCD and cisplatin over 48 h incubation period against A549 lung cancer cell lines are illustrated in Fig. 5b. The percentage cell viability during 24 h incubation period of CuONPs@βCD and cisplatin are also depicted in Fig. 5b for comparison. CuONPs@βCD and cisplatin over 48 h incubation period at 10 µg/mL demonstrated 73.04% ± 0.83 and 32.42% ± 0.51 cell viability; while at 50 µg/mL, cell viability dropped to 6.42% ± 0.64 and 0.18% ± 0.32, and further decreased almost up to 0.03% ± 0.02 and 0% ± 0.01 at 100 µg/mL, respectively. Furthermore, the IC_50_ values obtained for CuO NPs (24 h), cisplatin at 24 and 48 h, and CuONPs@βCD at 24 and 48 h of incubation were 43.36 ± 0.05 µg/mL, 9.9 µg/mL, 7.02 µg/mL, 41.06 ± 0.05 µg/mL, and 19.46 µg/mL, respectively. Thus, CuONPs@βCD demonstrate a dose-dependent effect on the A549 lung cancer cell line. Further it showed improved IC_50_ value of 19.46 µg/mL at 48 h incubation period.

Additionally, the impact of CuONPs@βCD and cisplatin were evaluated by comparing it with non-tumoral HEK293 cells (normal, embryonic kidney-derived) through MTT assay, as illustrated in Fig. 5c. CuONPs@βCD and cisplatin tested during 24-h incubation period against HEK293 normal cells at 10 µg/mL exhibited 92.27% ± 1.89 and 62.36% ± 0.39 cell viability; at 50 µg/mL, cell viability reduced to 49.42% ± 1.26 and 0.33% ± 0.06, and further decreased almost up to 0% ± 0.01 and 0% ± 0.01 at a higher concentration of 100 µg/mL, respectively. Similarly, CuONPs@βCD and cisplatin (48 h incubation period) at a lower concentration (10 µg/mL) displayed 75.19% ± 0.11 and 32.42% ± 0.51 cell viability; at 50 µg/mL, cell viability reduced to 7.32% ± 0.30 and 0.18% ± 0.32, and further decreased almost up to 0% ± 0.23 and 0% ± 0.01 at a higher concentration (100 µg/mL), respectively. The IC_50_ values obtained for CuONPs@βCD at 24 and 48 h, and cisplatin at 24 and 48 h incubation against HEK293 non-tumoral cells were 50 µg/mL, 22 µg/mL, 13.79 µg/mL, and 8.6 µg/mL, respectively. Therefore, these results reveal convincing evidence for the cytotoxic effect of CuONPs@βCD against A549 cancer cell line while having less adverse effects on HEK293 normal cell line.

The SI values calculated through Eq. (1) for cisplatin and CuONPs@βCD at 24 and 48-h incubation periods were depicted in Table 1. This SI value of CuONPs@βCD revealed improved bioavailability and cytotoxic potential as reported in the literature for similar NPs^56^. Additionally, the comparison of anticancer potential of other CuO NPs reported in literature against lung cancer cell is demonstrated in Table 2. From Table 2, it can be concluded that CuONPs@βCD displayed better cytotoxicity.

**Table 1:** The IC_50_ and SI values of cisplatin and CuONPs@βCD.

Material	A549		HEK293		SI	
	IC_50_ (µg/mL)		IC_50_ (µg/mL)			
	24 h	48 h	24 h	48 h	24 h	48 h
Cisplatin	9.9	7.02	13.79	8.6	1.39	1.22
CuONPs@βCD	41.06 ± 0.05	19.46	50	22	1.21	1.13

**Table 2:** Comparison of anticancer potential of various copper oxide NPs.

Material	IC_50_ (µg/mL)	References
CuO NPs	43.36 ± 05	^52^
CuO NPs	42.86 ± 0.05	^57^
CuO NPs	200.0 ± 0.05	^58^
CuO NPs	62.5 ± 0.05	^59^
CuO NPs	147.11 ± 1.13	^60^
CuO NPs	50.0 ± 0.05	^61^
CuONPs@βCD	41.06 ± 0.05	Proposed work

**Fig. 6:**
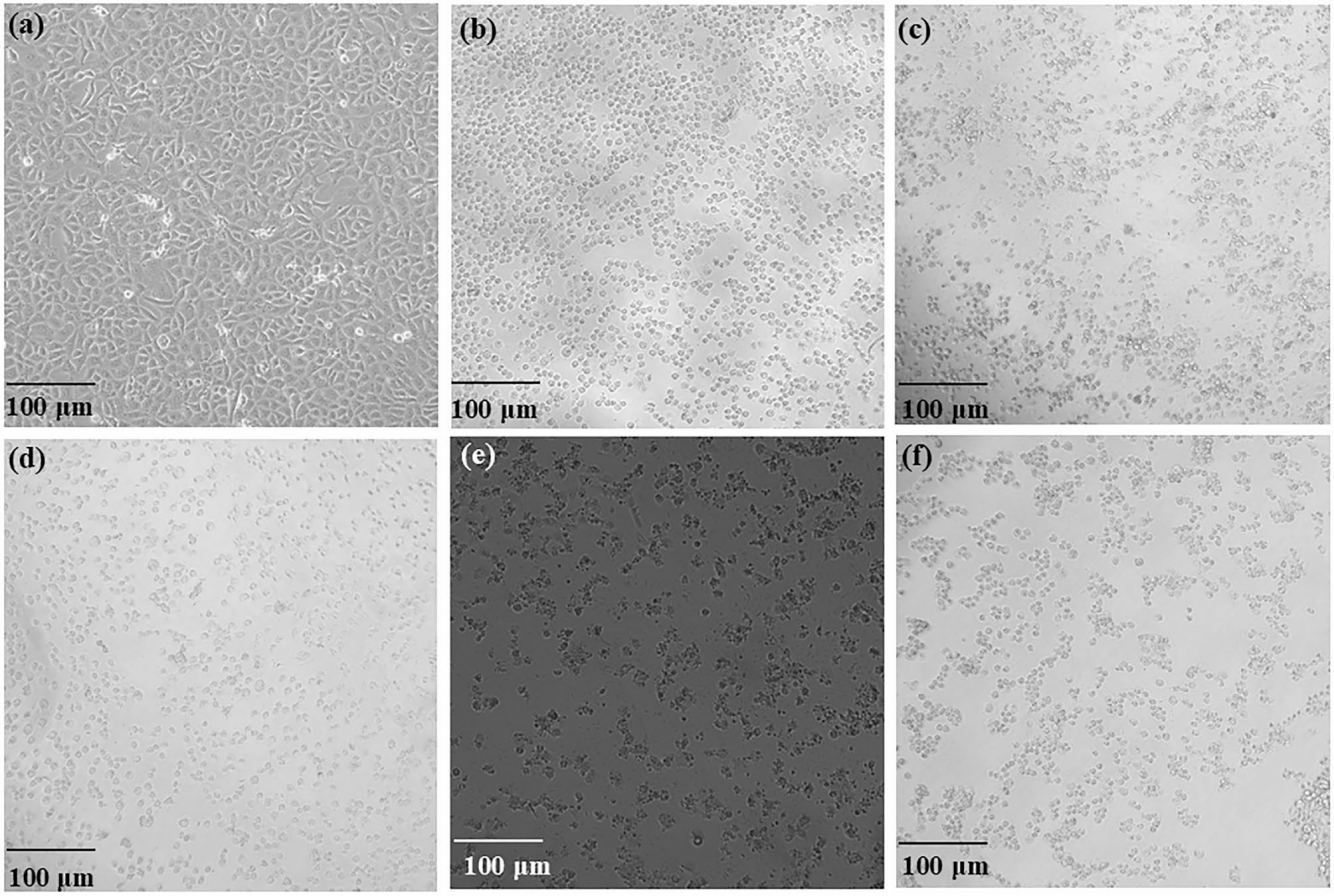
Morphological appearance of A549 hypotriploid alveolar basal epithelial cancer cell in phase contrast microscopy for (**a**) untreated cells and treated cells with (**b)** CuO NPs (24 h), (**c**) cisplatin (24 h), (**d**) cisplatin (48 h), (**e**) CuONPs@βCD (24 h), and (**f**) CuONPs@βCD (48 h).

The phase contrast microscopic images of the A549 lung cancer cell untreated and treated with CuO NPs, cisplatin (24 and 48 h), and CuONPs@βCD (24 and 48 h) are presented in Fig. 6a-f. The test medication of CuONPs@βCD has triggered numerous morphological alterations, including shape transformation, obstruction of cell communication, and chromatin condensation against A549 cancer cell which ultimately resulted in cell death^58,62,63^.

### Apoptosis study

**Fig. 7:**
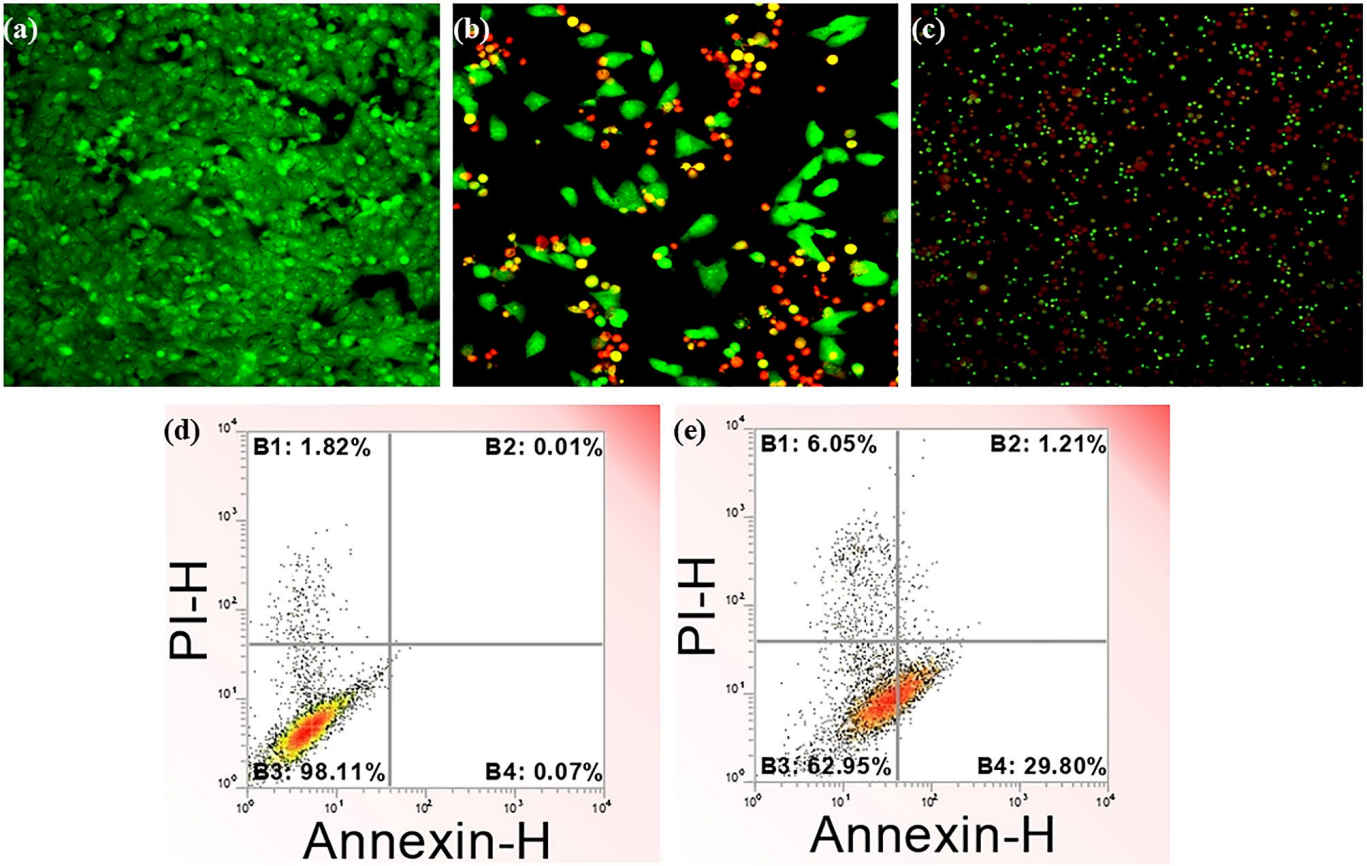
Fluorescent image of A549 (**a**) untreated cell line and treated cells with (**b**) cisplatin and (**c**) CuONPs@βCD. Annexin V-FITC/PI double staining of the (**d**) control (untreated), and after (**e**) CuONPs@βCD treated with IC_50_ concentration (24 h) in A549 cell. Scale bar: (**a** & **b**) 20 μm and (**c**) 100 μm.

In this study, the evaluation of apoptosis with cisplatin and CuONPs@βCD was conducted by applying AO/EB staining coupled with fluorescence. This technique enabled the investigation of how these test medications triggered cell death mechanisms. The staining procedure involved using AO and EB, each with distinct fluorescence properties. AO, known for its green fluorescence, can penetrate both viable and non-viable cells, providing a comprehensive view of the cell population. On the other hand, EB emits red fluorescence in cells that have lost membrane integrity. Apoptotic cells typically appear green due to AO staining, showing distinct morphological changes such as the formation of apoptotic bodies and cell blebbing, indicative of programmed cell death^64,65^. The differentiation between living, apoptotic, and necrotic cells was clearly observable under the fluorescence microscope. As shown in Fig. 7a–c, untreated A549 cells revealed constant green fluorescence with unique morphologies. Nonetheless, the A549 cells treated with cisplatin and CuONPs@βCD exhibited a red-orange fluorescence profile indicative of apoptotic cell death, highlighting features like fragmented chromatin and the presence of apoptotic bodies. This change in fluorescence behavior strongly suggests that CuONPs@βCD administered to the cells were primarily responsible for inducing apoptosis at concentrations corresponding to IC_50_. Therefore, we believe that our findings underscore reasonable and convincing evidence for the anticancer activity of synthesized CuONPs@βCD.

The apoptotic mechanism plays a crucial role in maintaining homeostasis and facilitating morphological and biochemical alterations in cells. Using a flow cytometer, the Annexin V (a calcium-dependent phospholipid-binding protein) and propidium iodide (PI) staining procedures are employed to determine the percentage of apoptosis induced by CuONPs@βCD in A549 cells. Annexin V, a calcium-dependent phospholipid-binding protein, exhibits an increased affinity for phosphatidylserine on the cytosolic side of the cell membrane. Staining analysis reveals four distinct groups of cells following Annexin V-FITC and PI staining: live cells, necrotic cells, early apoptotic cells, and late apoptotic cells^32,66^. The quantitative experiment demonstrated the extent of cancer cell death in A549 lung cancer cells, as assessed by flow cytometry using FITC Annexin-V and PI staining. Figure 7d and e present representative dot plots of Annexin V-FITC/PI staining, illustrating the percentages of apoptotic cells. The top right quadrant represents dead cells in the late stage of apoptosis (necrotic cells), the bottom right quadrant indicates cells undergoing apoptosis, and the bottom left quadrant denotes viable cells. Untreated A549 cells (Fig. 7d) exhibited a lower percentage of early apoptotic cells and late apoptotic cells at 0.07% and 0.01%, respectively, compared to CuONPs@βCD-treated cells, indicating that apoptosis was induced. Moreover, A549 cells treated with CuONPs@βCD exhibited a higher percentage of early apoptotic cells (29.80%) and late apoptotic cells (1.21%) (Fig. 7e). These findings demonstrated that CuONPs@βCD displayed greater cytotoxic efficacy compared to the control (untreated cells), leading to the induction of cell apoptosis.

### ROS detection with carboxy-H_2_DCFDA, anticancer mechanism, and MMP evaluation

The cytotoxic action of CuONPs@βCD is caused by the generation of ROS leading to cell death.

**Fig. 8:**
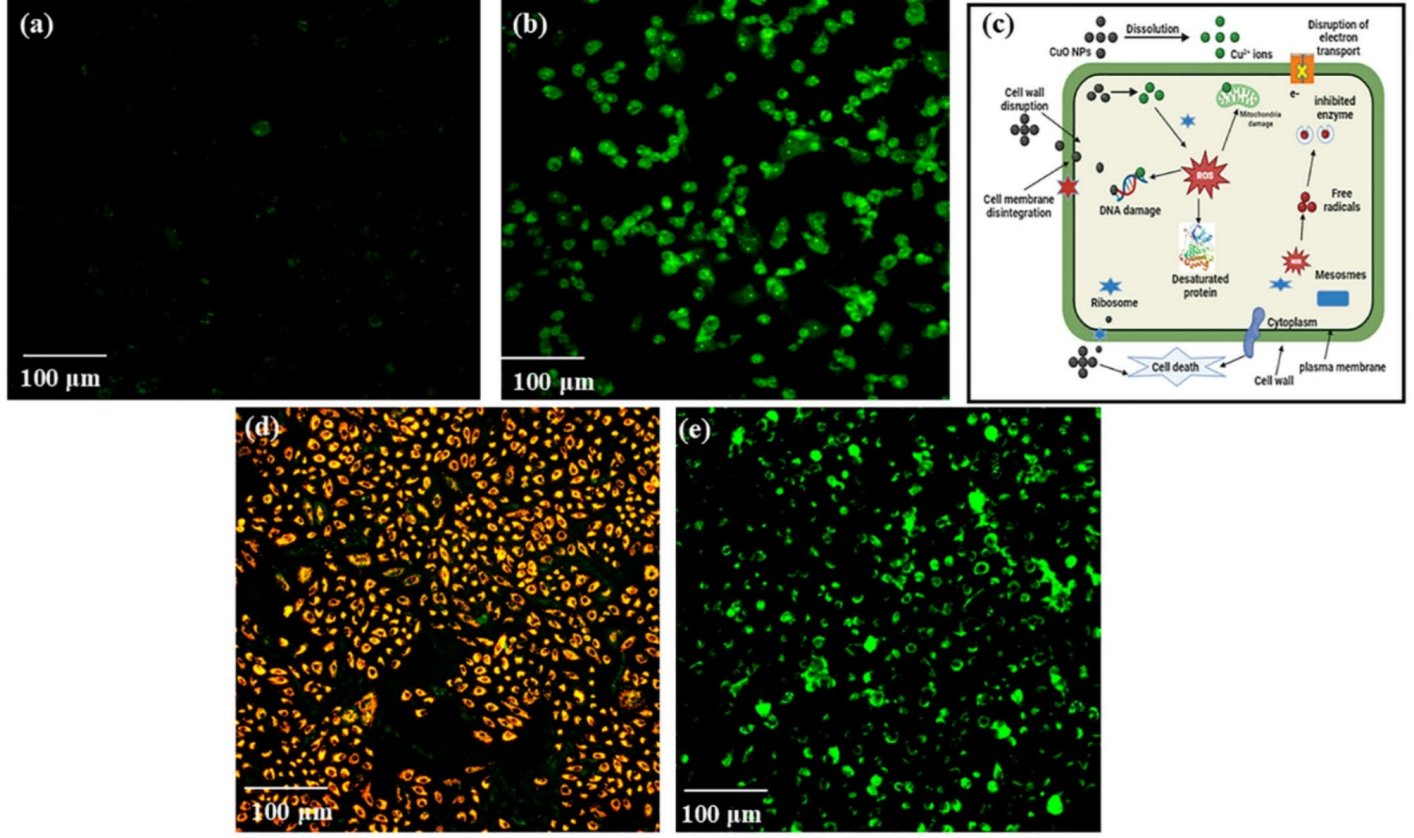
Intracellular ROS level in the A549 cells after treatment of (**a**) control, (**b**) CuONPs@βCD, (**c**) proposed anticancer mechanism of CuONPs@βCD, & MMP level in the A549 cells after treatment of (**d**) control, and (**e**) CuONPs@βCD. The data are presented as the means ± SD. *n* ≥ 3 regions with a total of 1500–2000 cells analyzed.

The findings indicate that CuONPs@βCD may induce cell death via apoptosis. The level of ROS generation was assessed using carboxy-H_2_DCFDA to ascertain if the apoptosis triggered by synthesized CuONPs@βCD is mediated by ROS formation or not^29^. Carboxy-H_2_DCFDA is non-fluorescent; however, it undergoes green fluorescent transformation when oxidized in the presence of ROS. The fluorescence microscopy analysis demonstrated that CuONPs@βCD treated cells produced an increase in fluorescence, which suggests the generation of ROS, in contrast to the control cells, which did not generate any ROS (Fig. 8a, b). Therefore, we hypothesize that elevated ROS levels can cause free radical attacks on membrane phospholipids, resulting in loss of MMP. The up regulated ROS level in cancer cells affects mitochondrial activities and plays a vital role in apoptosis induction^67^. Moreover, ROS-induced HepG2 cell death from hyperthermia using magnetic NPs has been reported in the literature^68^. The anticancer mechanism of CuONPs@βCD is demonstrated in Fig. 8c. CuO NPs engage with cancer plasma membranes and activate NP invagination via endocytosis, allowing NPs to penetrate the intracellular compartment^40^.

∆ψm is a crucial metric of mitochondrial function that indicates cancer cell health^30^. The lipophilic cationic JC-1 is an effective probe of vital mitochondria, accumulating primarily in the inner mitochondrial membrane^69^. In healthy cancer cells, JC-1 penetrates energized mitochondria and forms aggregate that alter the fluorescent properties of the JC‐1 dye. Unhealthy or apoptotic cells exhibit reduced ∆ψm. At elevated ∆ψm, JC-1 forms aggregate and emit red fluorescence; conversely, at reduced ∆ψm, JC-1 exists as a monomer and emits green fluorescence^70^. As shown in Fig. 8d, e, the mitochondria were stained red (Fig. 8d), and after treatment of the cells with CuONPs@βCD for 30 min, emitted green fluorescence (Fig. 8e). The CuONPs@βCD treated with JC-1 exhibited a gradual decline in red J-aggregate fluorescence and an increase in the cytoplasmic dispersion of green monomer fluorescence in cells. These results suggest that synthesized CuONPs@βCD have significant anticancer activity.

### The significance of size, shape, crystal structure, surface morphology, surface charge, and chemical composition on the antimicrobial and anticancer properties of CuONPs@βCD

The size and shape of NPs are essential considerations in antimicrobial evaluation^21,71^. CuONPs@βCD demonstrated significant antibacterial efficacy against *E. coli* and *S. aureus*, attributed to its small crystallite size of 9.36 nm, particularly along the maximum intensity planes (200) and (002) within the monoclinic crystal structure of copper in CuONPs@βCD. The antifungal activity of CuONPs@βCD exhibited comparable effect to those of the control. The spherical morphology of CuONPs@βCD offers an extensive surface area that improves interactions with bacterial and cancerous cells, disrupting membrane integrity, inducing cellular leakage, and ultimately resulting in cell death. CuONPs@βCD effectively target cancer cells through endocytosis, attributed to their small particle size of 12 nm and spherical form, demonstrating significant anticancer activity^72^. Furthermore, CuONPs@βCD facilitate the liberation of copper ions and the production of ROS. The particle size of CuONPs@βCD is also crucial in anticancer research. Cytotoxicity evaluations on A549 lung cancer cells revealed that CuONPs@βCD, characterized by a particle size of 12 nm, exhibited dose-dependent efficacy against the cancer cell line.

The ZP plays a crucial role in determining the antimicrobial and anticancer properties of test medications. An absolute ZP of 5.73 mV facilitates cellular uptake through electrostatic interactions with the cell membrane. Additionally, it enhances interactions with particular cellular targets, thereby promoting the release of copper ions, which is a crucial mechanism for their therapeutic efficacy. ZP significantly affects the biodistribution of NPs, thereby influencing their overall efficacy.

Furthermore, through Fenton-type processes, the Cu^2+^ oxidation state catalyzes antibacterial activity by producing ROS such as •OH that target bacterial cell membranes, proteins, and DNA, hence increasing antibacterial efficacy. Cu^2+^ complexes in cancer therapy can disrupt cellular functions by interacting with DNA and proteins. Additionally, the role of oxygen species, including singlet oxygen (^1^O_2_), is essential in antibacterial and anticancer activities, as they can cause oxidative damage to bacterial membranes and proteins^24^. Superoxide (O_2_^∸^) produced in biological systems can dismutate into H₂O₂, which may then generate •OH in the presence of copper, thereby inducing oxidative stress in bacterial and cancerous cells resulting in damage to their internal structures.

### Optical signature of CuONPs@βCD in double distilled water

Since these CuONPs@βCD exhibited improved anticancer and antimicrobial properties, we further explored their relevance for radiation therapy. Therefore, spectral transmittance of CuONPs@βCD in double distilled water (DDW) was evaluated using a UV-Vis spectrophotometer (Shimadzu UV-1800) in the wavelength range of 200–1100 nm. Measurements were taken for different concentrations ranging from 0.006 to 0.08 g/L. The measurement was conducted at the ambient temperature, and the spectral transmittance is depicted in supporting information Fig. S1. This result reveals that CuONPs@βCD in DDW exhibits high transmittance (almost 100%) within the wavelength range of 670–900 nm. However, it shows low transmittance at 400 and 970 nm, indicating that CuONPs@βCD in DDW has strong absorption at these specific wavelengths.

Typically, transmittance is represented as a percentage and defined as the ratio of transmitted light intensity to incident light intensity^73^. Thus, using Beer-Lambert’s rule, we can determine the extinction coefficient in terms of intensity of incident light traveling through a medium containing CuONPs@βCD.

**Fig. 9:**
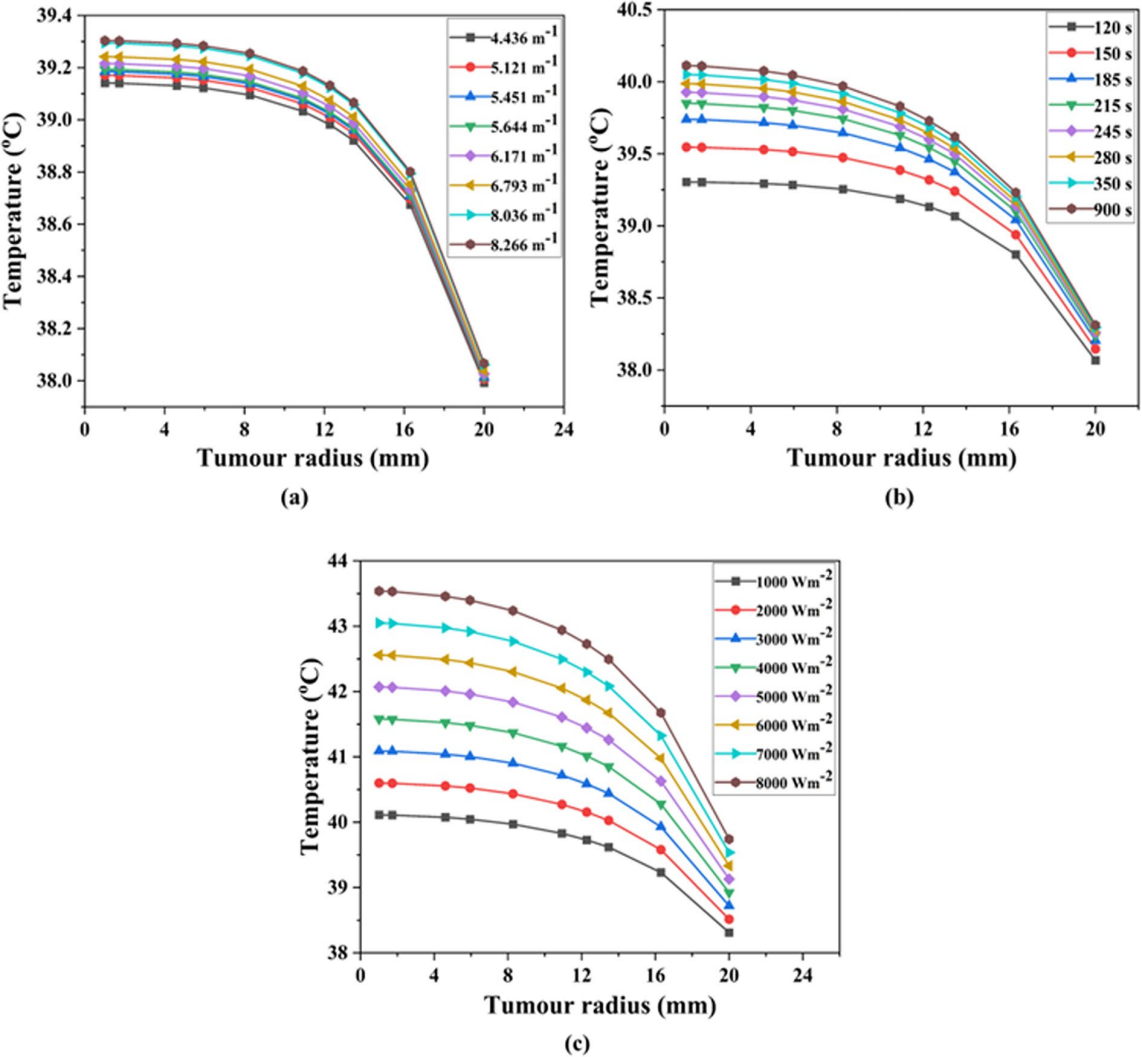
Variation in temperature (°C) at the bottom (*Z*) of 0 mm of lung tumorous tissue. By varying (**a**) extinction coefficient, (**b**) time, and (**c**) incident flux.

**Fig. 10:**
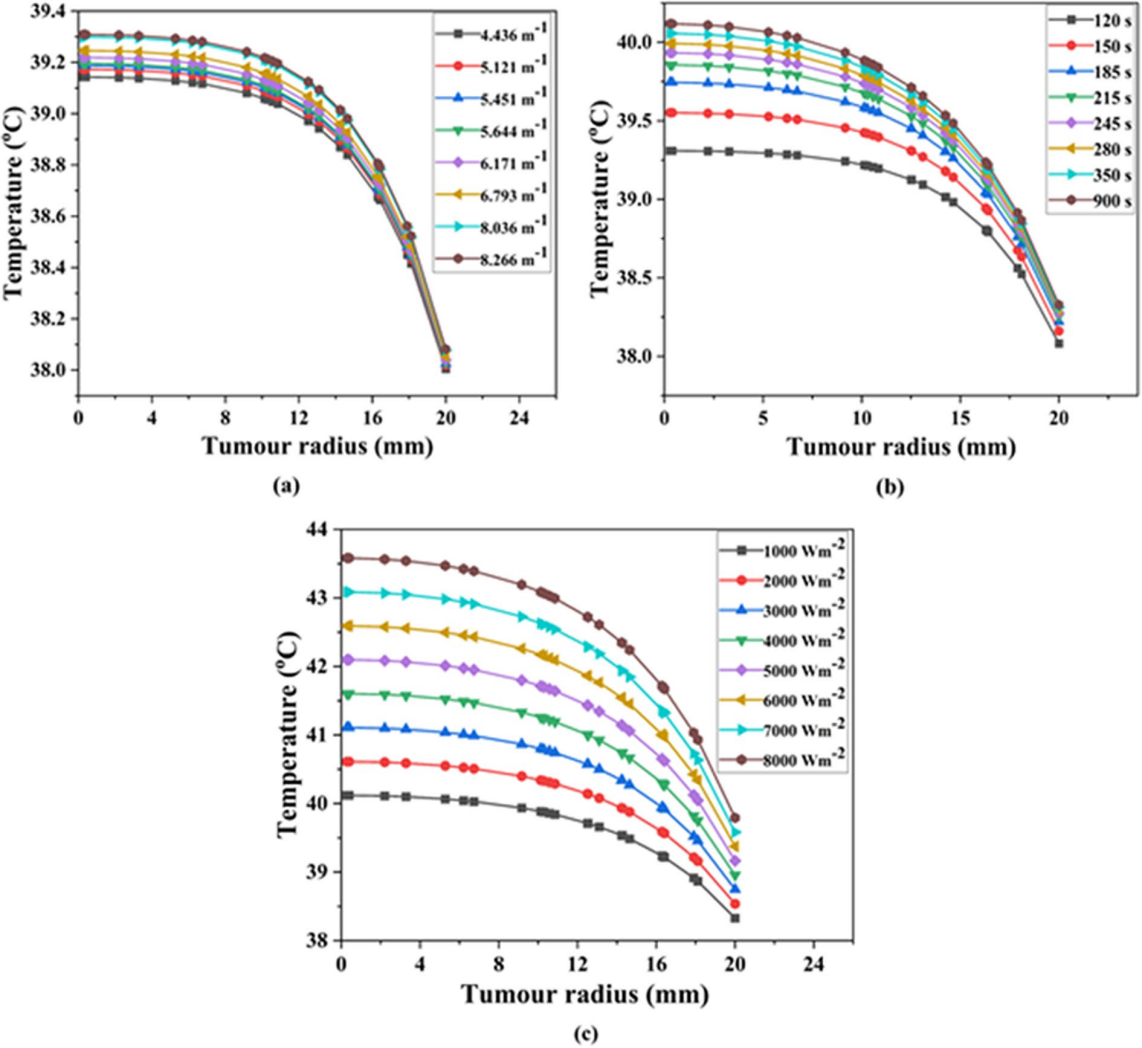
Variation in temperature at the center of the tumor depth (*Z*) of 5 mm. By varying (**a**) extinction coefficient, (**b**) time, and (**c**) incident flux.

**Fig. 11:**
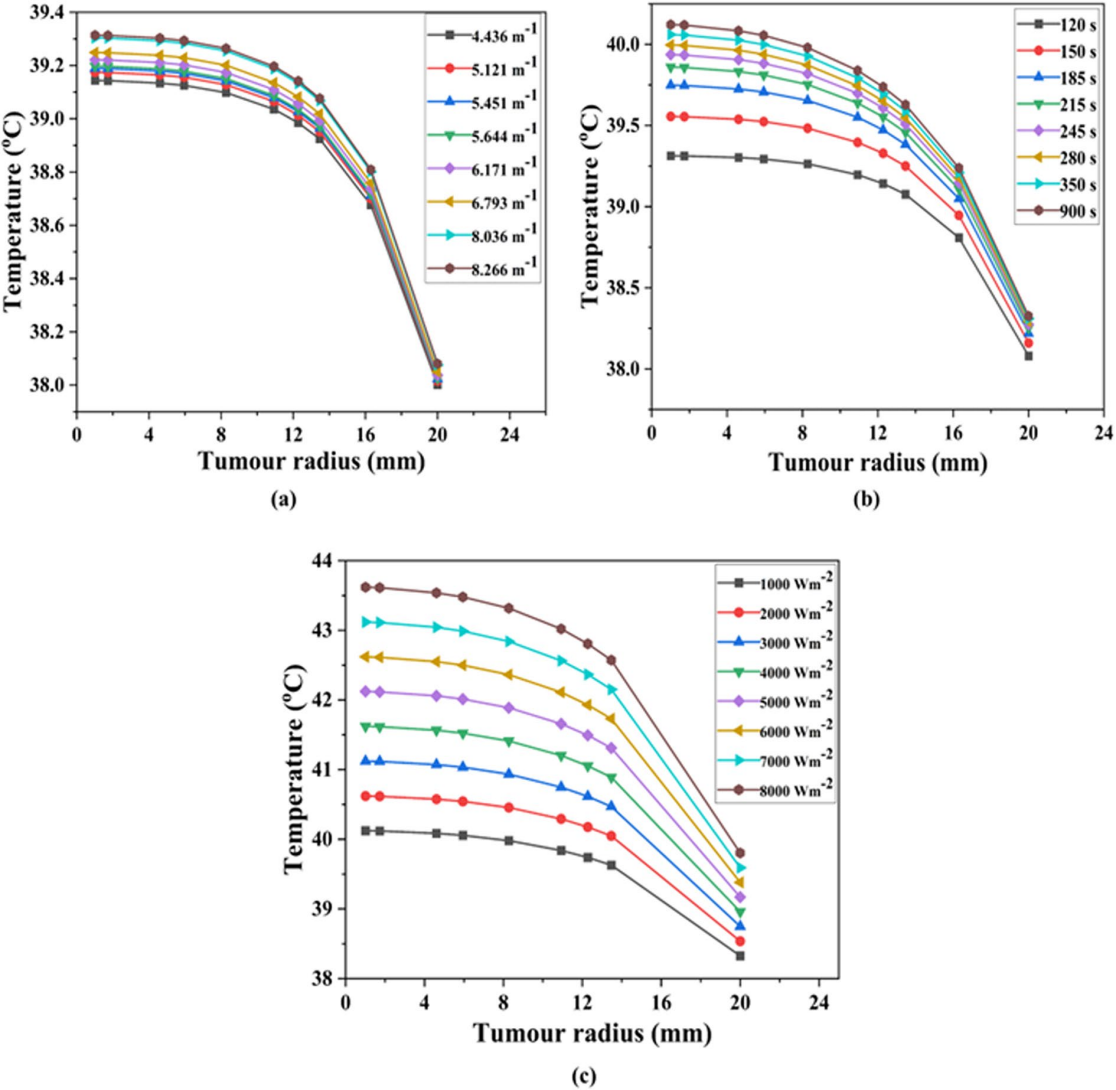
Variation in temperature at the top surface of the lung tumor depth (*Z*) of 10 mm. By varying (**a**) extinction coefficient, (**b**) time, and (**c**) incident flux.

Studies have revealed that a slight increase in tumor temperature (hyperthermia) makes cancer cells more sensitive to radiation and chemotherapy^74^. So, in this section, the results from the COMSOL Multiphysics modelling are put together with the general form partial differential equation (PDE) and heat transfer mathematical formulations of the temperature profiles and distributions. Since both the body and the lung have a normal temperature of about 37 °C^25^, an average temperature of 37 °C was utilized as the initial condition in bioheat transfer for analyzing the spatiotemporal temperature field. The required temperature to thermally damage the lung cancerous tissue is 42 °C^75^. Figures 9, 10 and 11 show the evaluated temperature fields under various conditions. Also, we looked at how the temperature changed along the tumor radius at three different depths: the tumor-tumor surface (*Z* = 0 mm), the tumor center (*Z* = 5 mm), and the tumor-healthy tissue boundary (*Z* = 10 mm).

### Temperature distribution at the bottom of tumor surface- case I

Case I represents the situation in which distribution of temperature happens at the bottom of tumor surface embedded with CuONPs@βCD, as illustrated in Fig. 1. Figures 9a–c assessed and illustrated the temperature distribution field for Case I type.

Understanding the extinction coefficient, time, and incident flux in a thermal therapy study is important for correctly modeling and analyzing how heat transfers through lung tissues. The extinction coefficient is a parameter that describes the attenuation of a wave as it propagates through a medium^76^. It is associated with the absorption and scattering properties of the material. The absorption coefficient determines the amount of energy (usually light or electromagnetic radiation) absorbed per unit distance as it passes through a material^77^. The incident flux refers to the amount of energy that incidents the lung tissue’s surface per unit area and per unit time^78^. In thermal therapy, time refers to the duration of administering the therapeutic energy to the lung tissue^79^.

Figure 9a demonstrated how changing the extinction coefficient can increase the required temperature while keeping the time (120 s) and incident flux (1000 Wm^− 2^) constant. According to Fig. 9a, the temperature at the tumor surface ranged from 39.10 to 39.30 °C. We observed the highest temperature of 39.30 °C at a tumor radius of 20 mm and a depth of 0 mm within the lung tumor at an extinction coefficient of 8.266 m^− 1^. Figure 9b showed that the target temperature rises as the irradiation time changed when the extinction coefficient (8.266 m^− 1^) and incident flux (1000 Wm^− 2^) resided the same. According to Fig. 9b, the temperature at the tumor surface ranged from 39.30 to 40.10 °C, with the highest temperatures observed at a tumor radius of 20 mm and a depth of 0 mm at 900 s. Once the incident flux was changed while the extinction coefficient (8.266 m^− 1^) and time (900 s) stayed the same, as shown in Fig. 9c, the temperature at the bottom of tumor location reached 43.50 °C with an incident flux of 8000 Wm^− 2^. Figure 9a–c demonstrated the temperature increase in the lung tumor tissue at a depth of 0 mm, ranging from 39.10 to 43.50 °C.

### Distribution of temperature at the center of the tumor area – case II

Case II represented the situation when temperature distribution occurs at the tumor center (Z = 5 mm), as shown visually in Fig. 1. Figure 10a–c displayed the examination of temperature field within lung tumorous tissue in this temperature distribution instance. Figure 10a further demonstrated that altering the extinction coefficient can increase the required temperature while maintaining the same incident flux (1000 Wm^− 2^) and time (120 s). In this case, the surface temperatures of the lung tumor also varied between 39.10 and 39.30 °C, as shown in Fig. 10a. The highest temperatures at a depth of 5 mm within the lung tumor, with an extinction coefficient of 8.266 m^− 1^ have been recorded. As the irradiation time changes, Fig. 10b showed that the target temperature rises when the extinction coefficient (8.266 m^− 1^) and incident flux (1000 Wm^− 2^) resided the same.

Similarly, in this case, the temperature at the tumor surface varied between 39.30 and 40.10 °C, as indicated by Fig. 10b. The most significant temperatures at a depth of 5 mm at 900 s have been recorded. Further, by changing the amount of incident flux while keeping the extinction coefficient (8.266 m^− 1^) and time (900 s) constant, as represented in Fig. 10c, the temperature at the tumor center reached 43.50 °C, similar to case I at an incident flux of 8000 Wm^− 2^. Therefore, Fig. 10a–c illustrated the rise in temperature within the lung tissue at a depth of 5 mm, ranging from 39.10 to 43.50 °C, which is comparable to the pattern observed in case I.

### Temperature distribution at the top surface of the tumor—case III

Figure 1 illustrated Case III, a scenario where the temperature uniformly distributed at the top surface of the tumor (Z = 10 mm). We used the plot of temperature versus tumor radius along the depth of lung tissue (10 mm) to quantify the extent of thermal energy penetration, as shown in Fig. 11a–c. We assessed temperature variation along the tumor radius under three distinct conditions: varying extinction coefficient, time, and incident flux at a depth of 10 mm, as shown in Fig. 11a, b, and c, respectively.

Figure 11a demonstrated that changing the extinction coefficient can increase the required temperature while keeping the incident flux (1000 Wm^− 2^) and time (120 s) constant. In this case, the surface temperatures of the lung tumor varied between 39.10 and 39.31 °C, as indicated in Fig. 11a. The most substantial temperatures at a depth of 10 mm at 8.266 m^− 1^ extinction coefficient have been recorded. Furthermore, Fig. 11b showed that when the extinction coefficient (8.266 m^− 1^) and incident flux (1000 Wm^− 2^) remain the same, the target temperature rises as the irradiation time altered. Similarly, in this case, the temperature at the tumors top surface varied between 39.30 and 40.11 °C, as shown in Fig. 11b. Figure 11c illustrated how altering the amount of incident flux led to a temperature shift within the tumor surface from 40.09 to 43.63 °C, while maintaining the same extinction coefficient (8.266 m^− 1^) and time (900 s).

**Fig. 12:**
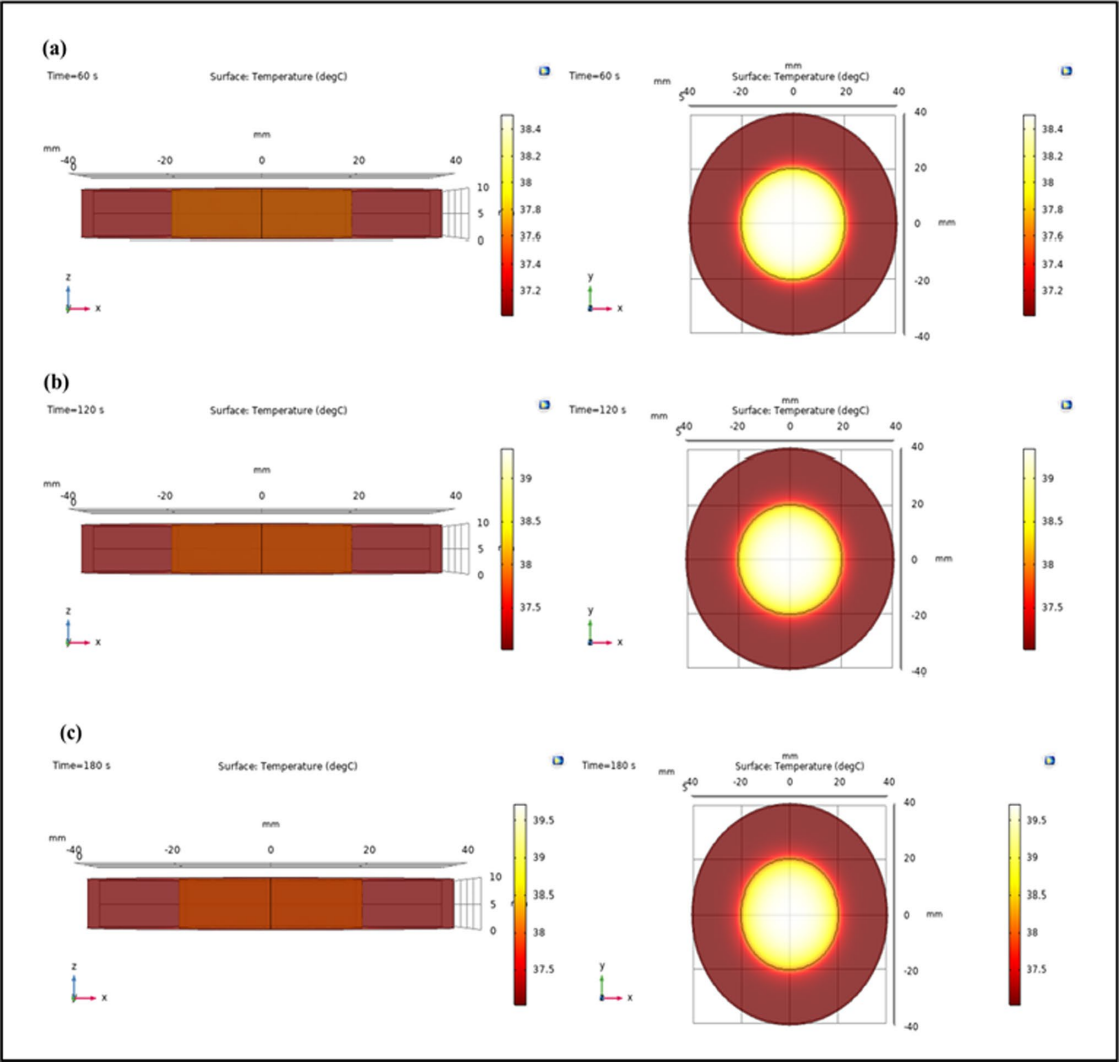
Surface temperature plot from COMSOL at the surface of lung tumor tissue in xz and xy planes at irradiation time of (**a**) 60 s, (**b**) 120 s, and (**c**) 180 s by varying the time.

A COMSOL surface temperature plot offers a visual representation of temperature distribution on a surface within a modeled domain. Understanding the spatial and temporal distribution of temperature within the modeled domain helps to interpret a surface temperature plot from COMSOL^80^. At several irradiation intervals, surface temperature plots in both the xz and xy planes allow us to understand the efficacy of the irradiation, the uniformity of heat distribution, and possible effects on both the tumor and surrounding tissues. The surface temperature plots of lung tumor tissue in xz and xy planes at various irradiation times by varying the time, extinction coefficient, and incident flux are depicted in Fig. 12 and supporting information Fig. S2 and S3, respectively. Changing the extinction coefficient makes the temperature rise from 37.80 to 38.50 °C on the surface of lung cancer tissue in the xz and xy planes during irradiation times of 60 and 120 s, as shown in the COMSOL surface temperature plot. Similarly, varying the time leads the temperature to rise from 37.80 to 38.50 °C, as evidenced by the surface temperature plot in the xz and xy planes during irradiation times of 60, 120, and 180 s. Likewise, when the incident flux is varied, the temperature rises from 38.50 to 40.00 °C, as demonstrated by the surface temperature plot during irradiation times of 60, 120, and 180 s.

In order to validate the present model, we have imitated the existing work of Soni et al.^34,81^ with the equation which we have utilized for the present study. Supporting information Fig. S4 demonstrates the radial variation of temperature at tumor depth (*Z*) of 5 mm by altering the time at 50, 100, and 150 s^34^. Also, the surface temperature plot from the supporting information Fig. S5 at the surface of tumor tissue in the xz and xy planes gives evidence of temperature rise by varying the irradiation time at 50, 100, and 150 s. Therefore, the results are nearly identical to those reported by Soni et al.^34^. According to Soni et al.., the temperature at the deeper tumor location T(10,5) remained unaffected at 37 °C exclusively, as seen in the reported literature from Fig. 5^34^. T(10,5) indicates that the tumor has a 10 mm radius and a 5 mm depth. Hence, it proves that our model matches the results of above-mentioned Soni et al. literature^34^.

Hyperthermia based clinical trials for cancer treatments have been reported in the literature involving metal oxide NPs to improve the effectiveness of chemotherapy and radiotherapy^16^. Here, βCD has been employed as a capping agent to reduce the nonspecific interactions of CuO NPs with normal cells and thereby positively influencing the IC_50_ values. Moreover, the distinct morphology, size and paramagnetic effect of CuONPs@βCD displayed promising antimicrobial activity which is essential for lung cancer. Hyperthermia has garnered significant attention due to its minimally invasive nature and potential for precise localization. Nanotechnology plays a crucial role in enhancing the effectiveness of thermal therapy. Therefore, our CuONPs@βCD present a promising avenue for anticancer therapy, either as standalone agents or as thermal therapeutic enhancers.

### Investigating the impact of tumor geometry on temperature distribution

**Fig. 13:**
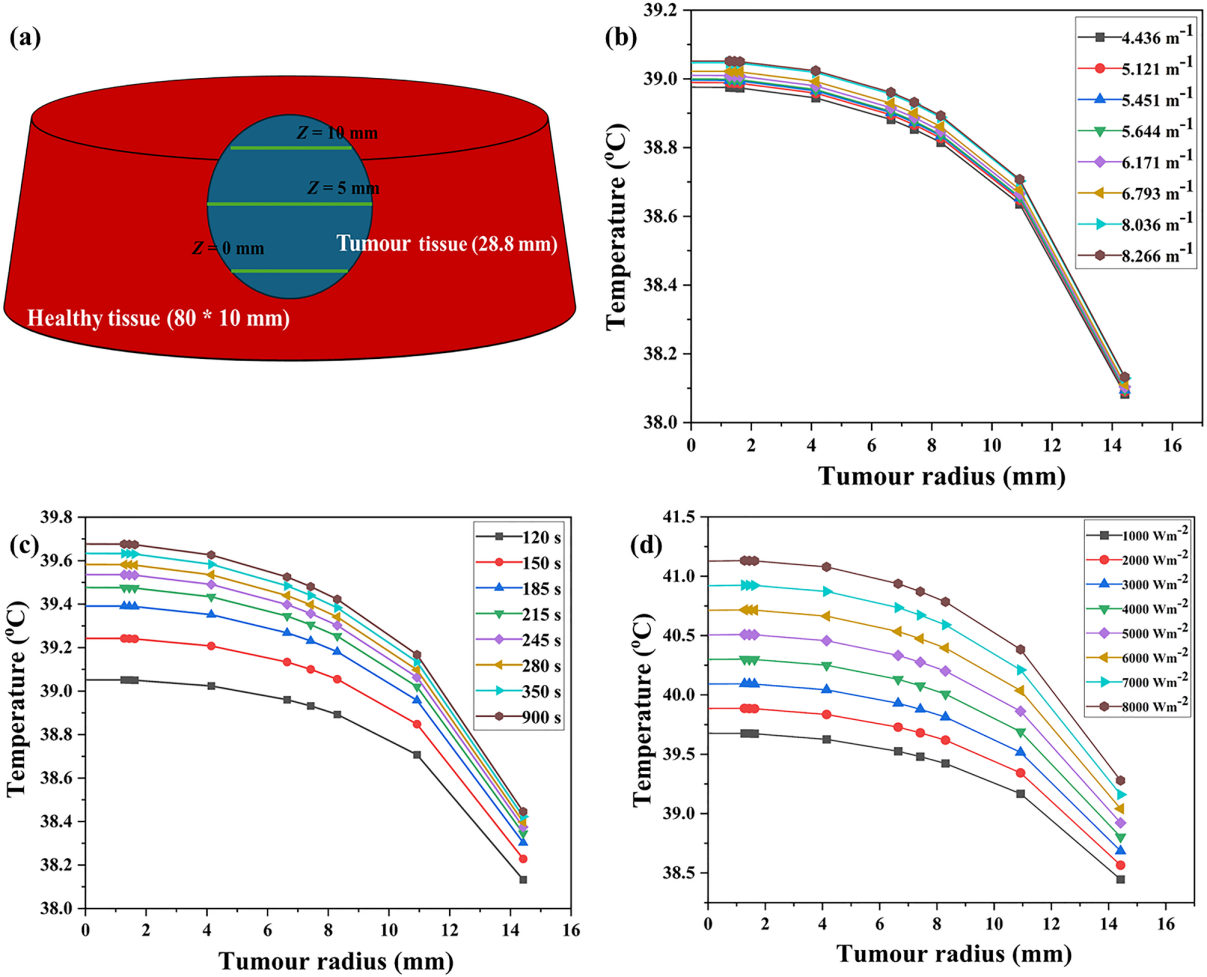
(**a**) Schematic illustration of spherical tumor geometrical region surrounded by healthy lung tissue. Three cases of spatial distribution of temperature along the depths (*Z* = 0, 5, and 10 mm) of tumor. Variation in temperature (°C) at the bottom (*Z*) of 0 mm of lung tumorous tissue. By varying (**b**) extinction coefficient, (**c**) time, and (**d**) incident flux.

The effect of different tumor shapes on temperature distribution must be considered. The way heat is dispersed and transported within a tumor and its surrounding tissues can be greatly impacted by its shape. This is because a tumors size, shape, and orientation in relation to the heat source can all affect the temperature distribution inside it. As a result, Fig. 13a showed a characteristic spherical lung cancerous tumor with a diameter of 28.8 mm (determined from the volume of the tumor cylinder) encircled by healthy cylindrical tissue with a diameter of 80 mm and a depth of 10 mm, illustrating the 3D hyperthermia process. In Case I, temperature distribution occurred at the bottom of the tumor surface embedded with CuONPs@βCD (Fig. 13a). Figure 13b–d depicted the temperature distribution field for Case I type.Figure 13b showed how altering the extinction coefficient increases the required temperature while keeping the time (120 s) and incident flux (1000 Wm^− 2^) constant. The temperature at the tumor surface varied from 38.97 to 39.05 °C, as illustrated in Fig. 13b. We recorded the maximum temperature of 39.05 °C within the lung tumor at an extinction coefficient of 8.266 m^− 1^, with a tumor radius of 14.4 mm. Figure 13c illustrated that the target temperature increased with variations in irradiation time, while the extinction coefficient (8.266 m^− 1^) and incident flux (1000 Wm^− 2^) remain constant. Figure 13c indicated that the temperature at the tumor surface varied between 39.05 and 39.68 °C, with peak temperatures recorded at a tumor radius of 14.4 mm after 900 s. When the incident flux was altered, maintaining the extinction coefficient at 8.266 m^− 1^ and time at 900 s, as illustrated in Fig. 13d, the temperature at the bottom of tumor attained 41.12 °C with an incident flux of 8000 Wm^− 2^. Figures 13b–d illustrated the temperature elevation in lung tumor tissue at a depth of 0 mm, with values ranging from 38.97 to 41.12 °C.

Case II depicted the circumstance in which temperature distribution occurred near the tumor centre (Z = 5 mm), as illustrated in Fig. 13a. Supporting information Figs. S6a-c showed an evaluation of the temperature field within the lung tumorous tissue in this temperature distribution instance. As demonstrated in Fig. S6a, the surface temperatures of the lung tumor ranged from 38.98 to 39.12 °C. The lung tumor has been found to have the maximum temperatures at a depth of 5 mm, with an extinction coefficient of 8.266 m^− 1^. The temperature at the tumor surface fluctuated between 39.11 and 39.69 °C as the irradiation time varied, as illustrated in Fig. S6b. The highest temperatures at a depth of 5 mm were observed at 900 s. Furthermore, by adjusting the incident flux, as shown in Fig. S6c, the temperature at the tumor center reached 41.91 °C at an incident flux of 8000 Wm^− 2^. As a result, Figs. S6a-c showed a rise in temperature within the lung tissue at a depth of 5 mm, ranging from 38.98 to 41.91 °C.

Additionally, Fig. 13a depicted Case III, a situation in which the temperature was consistently distributed across the top surface of the tumor (Z = 10 mm). Supporting information Fig. S7a illustrated that altering the extinction coefficient can elevate the necessary temperature while maintaining a consistent incident flux (1000 Wm^− 2^) and time (120 s). As shown in Fig. S7a, the surface temperatures of the lung tumor ranged from 38.96 to 39.10 °C. Furthermore, Fig. S7b demonstrated that when the extinction coefficient (8.266 m^− 1^) and incident flux (1000 Wm^− 2^) remain constant, the target temperature increased as the irradiation time altered. In this instance, the temperature at the top surface of the tumor ranged from 39.04 to 39.61 °C, as illustrated in Fig. S7b. Figure S7c demonstrated the effect of varying incident flux on the temperature of the tumor surface, resulting in a shift from 39.62 to 41.41 °C, while maintaining the same extinction coefficient (8.266 m^− 1^) and time (900 s).

Consequently, the results of comparison between a cylindrical lung tumor and a spherical geometrical region surrounded by healthy lung tissue indicated only a slight variation in distribution of temperature at the bottom, center, and top surface of tumor because of variations in extinction coefficient, time, and incident flux.

### Comparing model-predicted temperature distributions with experimental hyperthermia data

Experimental measurements and numerical findings were used to validate the model using hyperthermia data or to compare it to other modeling methods. The measurements were subsequently extended to tumor tissue, considering blood perfusion and metabolic heat generation. The proposed technique effectively confines heat to the nanoparticle-embedded region. The current investigation by Soni et al. involved embedding Au NPs in the upper region of the gel, which corresponds to a 4 mm thick tumor region exhibiting a uniform distribution of Au NPs. Agarose gel was made in a cylindrical configuration with a diameter of 40 mm and a depth of 4 mm. The central region of the gel, 12 mm in diameter, was embedded with Au NPs, while the surrounding area consisted of plain gel without NPs. The symbols, thermophysical properties, and parameter values were analyzed in accordance with the literature report by Soni et al.^34, 82^.

**Fig. 14:**
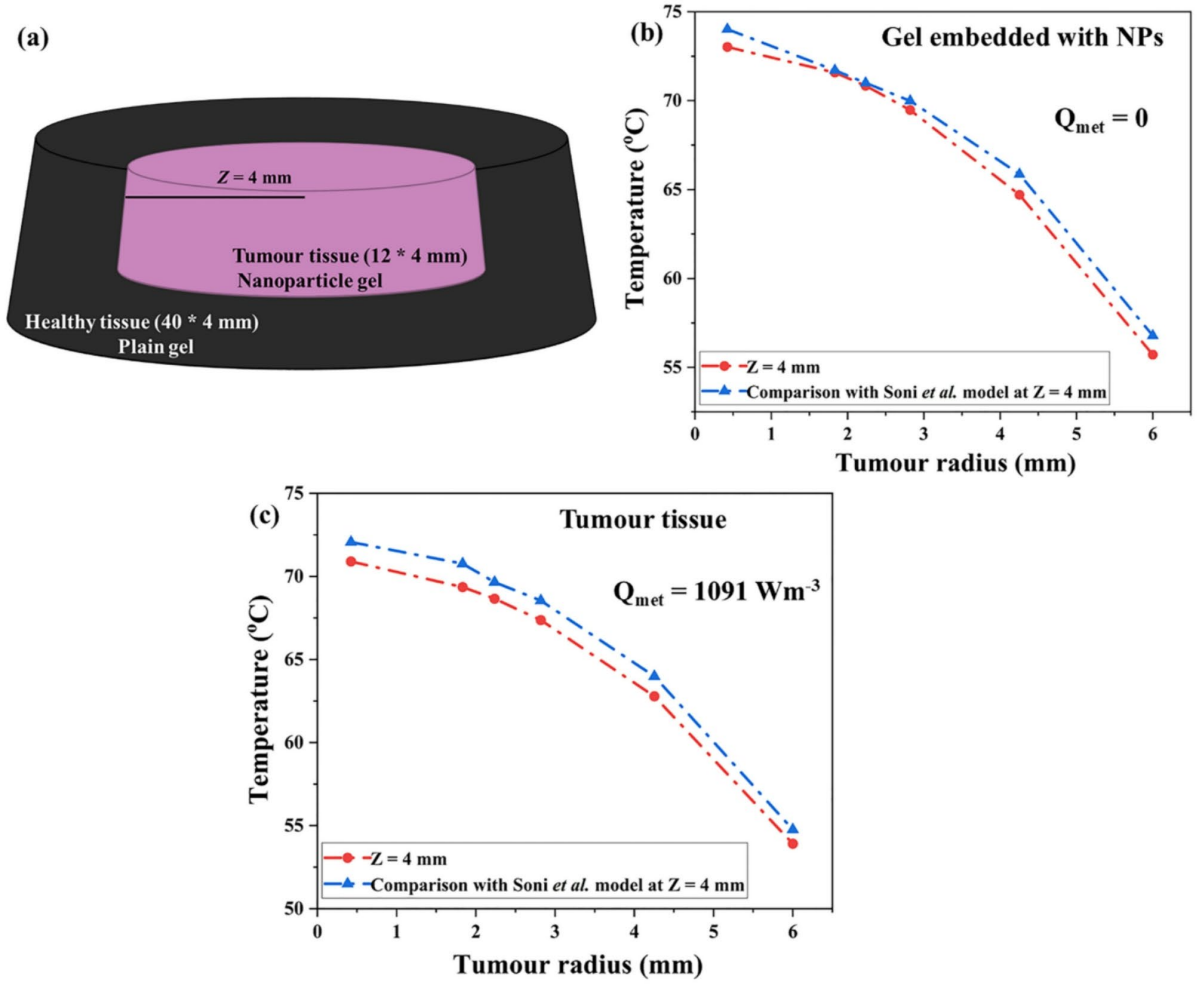
(**a**) Schematic illustration of a tumor location (nanoparticle gel) surrounded by healthy tissue (plain gel). (**b**) Temperature fluctuation was measured for a gel embedded with NPs. The incident flux of irradiation was kept as 25,000 Wm^− 2^ for 120 s. Blood perfusion and metabolic parameters were chosen as zero to simulate the temperatures within the gel. (**c**) Simulated spatial temperature within tumor tissue. Tumor blood perfusion was kept as 9.1 × 10^−4^ s^−1^, while tissue perfusion was 1 × 10^−3^ s^−1^. Metabolic heat generation within tumor and tissue was kept as 1091 Wm^− 3^. Spatial distribution of temperature along the depth *Z* = 4 mm of tumor. Also, comparison with Soni et al. model at Z = 4 mm using current modeling method.

Schematic illustration of a tumor location (nanoparticle gel) surrounded by healthy tissue (plain gel) was depicted in Fig. 14a. The observed temperatures for the nanoparticle-embedded gel in the agarose gel configuration depicted in Fig. 14a are presented in Fig. 14b along the radial location at 4 mm depth. Figure 14b indicated that a temperature of 73 °C was reached at the tumor center (Z = 4 mm) within the nanoparticle-embedded region. Moreover, the results of the comparison with Soni et al. model from Fig. 14b demonstrated only 1% variation in distribution of temperature through current modeling method.

The actual condition involves blood flow and metabolism within a tissue, which is absent in the agarose gel. The temperatures achieved in actual tissue will differ from those in gel, thereby affecting the spatial extent of heat confinement. As a result, the established numerical model, which was validated through temperature measurements on gel, was extended to account for real tissue conditions. Figure 14c showed corresponding temperatures for a tenfold increase in the tumor blood perfusion. Figure 14c denoted that a temperature of 71 °C was reached at the tumor center (Z = 4 mm) within the nanoparticle-embedded tissue region. Moreover, in this case the metabolic heat generation within tumor and tissue was kept as 1091 Wm^− 3^. It is seen from Fig. 14c that the variation in the maximum temperature for the real tissue versus gel is ~ 2 °C. Additionally, comparison with Soni et al. model from Fig. 14c showed only 1% variation in distribution of temperature through current modeling method.

It has been reported that the metabolic rate of tumor can be as high as 65,400 Wm^− 3^^82^. So, the temperature results were also evaluated with a metabolic heat of 65,400 Wm^− 3^ in the tumor. On account of higher tumor metabolism, the increase in the temperatures was evaluated as 0.7 °C. Furthermore, the fluctuation in temperature distribution at 1091 Wm^−3^ is not particularly significant. As a result, the experimental data matches with the theoretical modeling work. Thus, the comparison results with experimental hyperthermia data verified the variation in temperature distribution using the current modeling method.

Though, CuONPs@βCD shows potential in vitro anticancer, antimicrobial and thermal therapy studies, achieving accurate tumor targeting while reducing harmful effect to the surrounding healthy tissues presents a considerable challenge. Secondly, formulating strategies for targeted nanoparticle delivery and activating the tumor microenvironment are essential for enhancing therapeutic efficacy. Third, long-term studies are essential to evaluate the potential accumulation of NPs in the body and their prolonged effects on different organs. Finally, integrating this technology into clinical practice entails overcoming regulatory challenges and undertaking complex clinical trials to establish safety and efficacy in humans. Addressing these constraints is critical for maximizing the effectiveness of this method in fighting against lung cancer.

## Conclusion

In this study, we reported the synthesis and physicochemical properties of βCD-stabilized CuO NPs. CuONPs@βCD demonstrated spherical shape, reduced particle size, hydrodynamic size, polydispersity index, stability and surface area charge that are highly suitable for its anticancer and antimicrobial effects as evidenced from HR-TEM, DLS, and ZP studies. The CuONPs@βCD displayed antimicrobial activity as good as commercial drugs for various strains of bacteria and fungi. There is also strong evidence from the MTT assay that CuONPs@βCD can kill A549 lung cancer cells, with IC_50_ values of 41.06 ± 0.05 µg/mL and 19.46 µg/mL at 24 and 48-h incubation period, respectively. Furthermore, CuONPs@βCD suppressed A549 lung cancer cell through metastasis, enhanced ROS generation, apoptosis and altered Δψm level. Moreover, a low cytotoxic effect was observed in HEK293 normal cells, which demonstrated the biocompatibility of CuONPs@βCD. Additionally, we evaluated the suitability of CuONPs@βCD for thermal therapy using COMSOL Multiphysics. These NPs demonstrate good temperature distribution in tumor tissue which involves simulating the heat transfer within biological tissues that are useful for hyperthermia treatment in cancer cells. The heat was able to penetrate more effectively at the top surface of the tumor, resulting in an increase in temperature to a range of 39.30–43.63 C. It was found that an incident flux of 8000 Wm^− 2^ for 900 s and an extinction coefficient of 8.266 m^− 1^ for CuONPs@βCD were the best conditions for reaching a temperature of 43.63 C across the whole tumor area.

## Electronic supplementary material

Below is the link to the electronic supplementary material.

Supplementary Material 1

## Data Availability

The datasets used and/or analyzed during the current study available from the corresponding author on reasonable request.
