# Supplementary material for: Beta cyclodextrin stabilized cupric oxide nanoparticles assisted thermal therapy for lung tumor and its effective in vitro anticancer activity
Source: Sci Rep. 2025 Aug 7;15:28983. doi: 10.1038/s41598-025-96578-3 (PMC12332176; doi:10.1038/s41598-025-96578-3)
Supplement: Supplementary file 1 — Supplementary Material 1 [file 41598_2025_96578_MOESM1_ESM.doc]

**Supplementary Information**

**Beta cyclodextrin stabilized cupric oxide nanoparticles assisted thermal therapy for lung tumor and its effective in vitro anticancer activity**

Anakha D. Rajeeve^1,2^, Vyshnavi T. Veetil^1,2^, Sabarinathan Palaniyappan^3^, Ramasamy Yamuna^1,2^,*, Vishal Bhalla^4^,*

^1^Department of Chemistry, Amrita School of Physical Sciences Coimbatore, Amrita Vishwa Vidyapeetham, India.

^2^Bio-materials Chemistry Research Laboratory, Amrita School of Engineering Coimbatore, Amrita Vishwa Vidyapeetham, India.

^3^Center for biosystems and machines, King Fahd University of Petroleum and Minerals, Dhahran, Saudi Arabia.

^4^School of Energy and Environment, NICMAR University, Pune, Maharashtra, India.

Corresponding Author: [r_yamuna@cb.amrita.edu^1,2^](mailto:r_yamuna@cb.amrita.edu1,2)^,*^; [vishal.bhalla@pune.nicmar.ac.in^4^](mailto:vishal.bhalla@pune.nicmar.ac.in4)^,*^

**
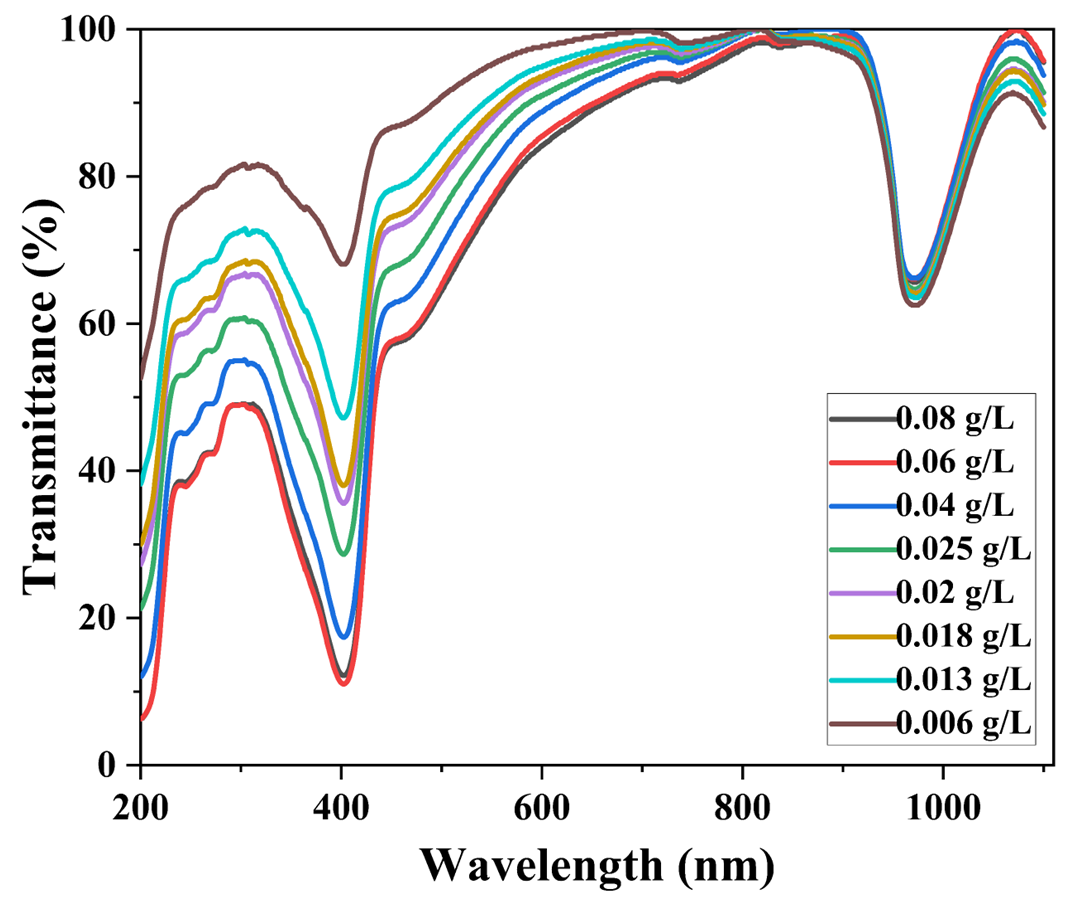
**

**Figure S1.** Transmittance plot of CuONPs@βCD in DDW (concentrations ranging from 0.006 to 0.08 g/L).

**
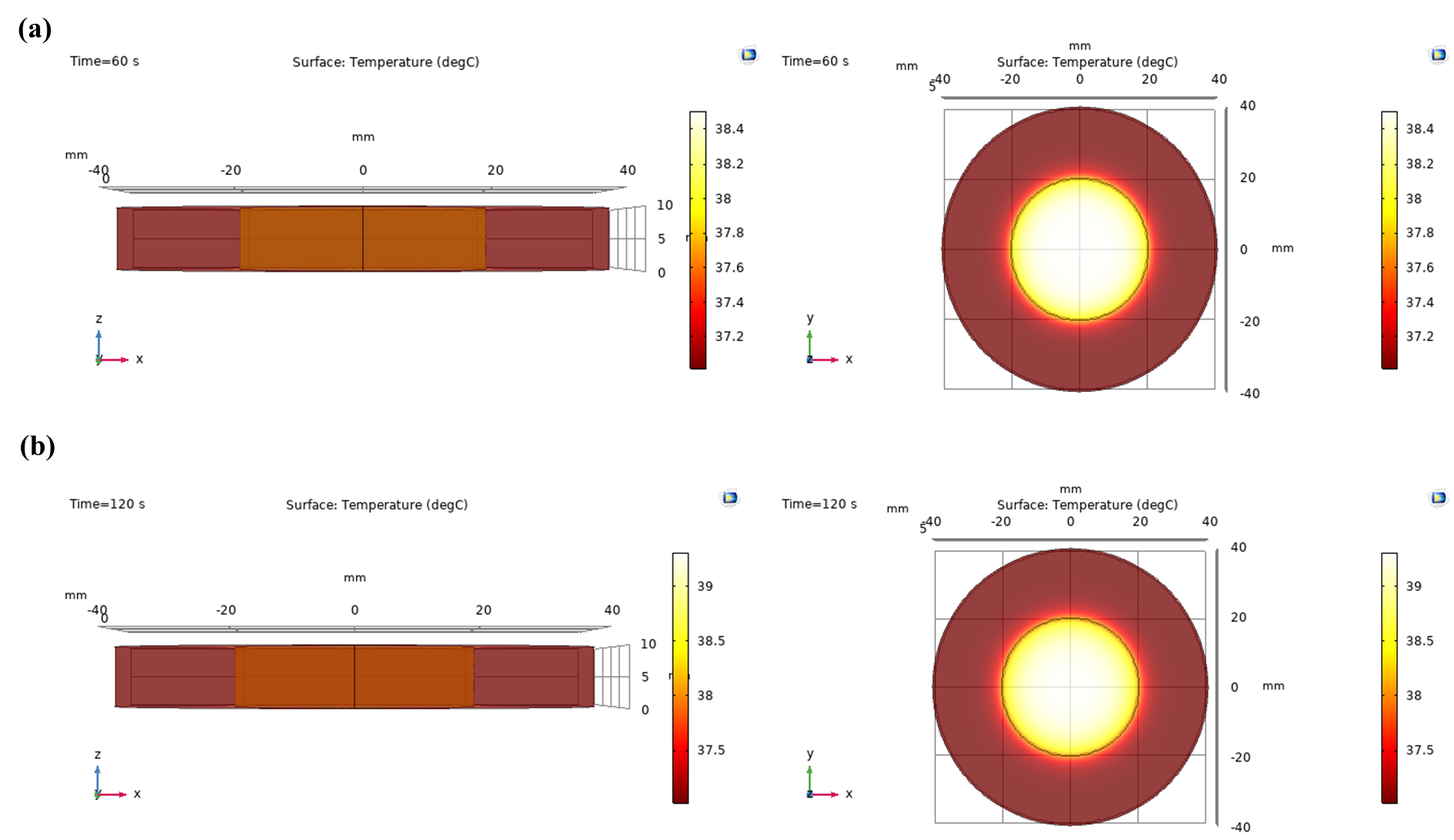
**

**Figure S2.** Surface temperature plot from COMSOL at the surface of lung tumor tissue in xz and xy planes at irradiation time of (**a**) 60 s and (**b**) 120 s by varying the extinction coefficient.


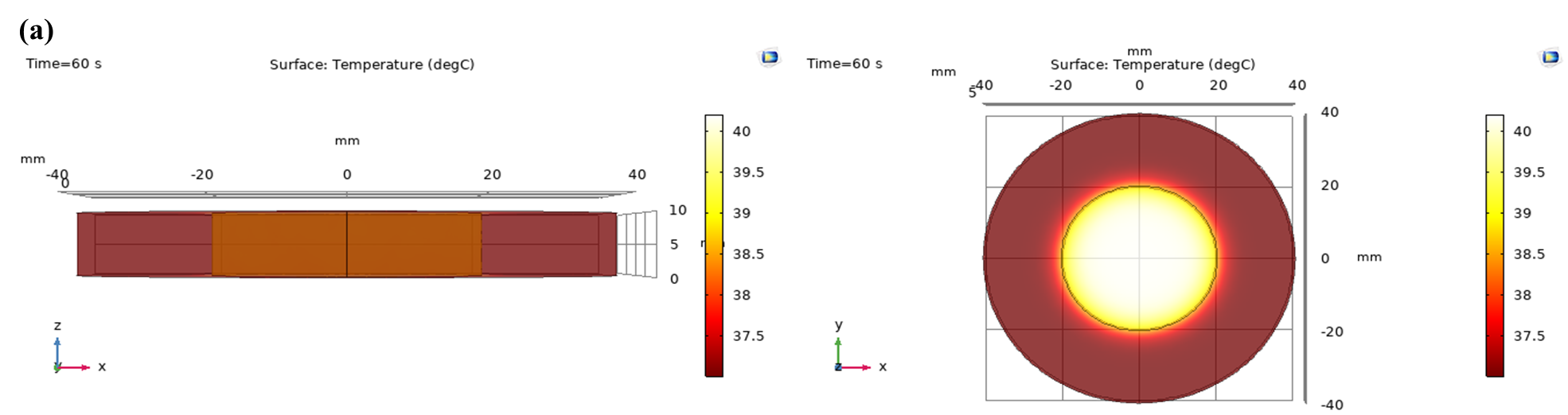

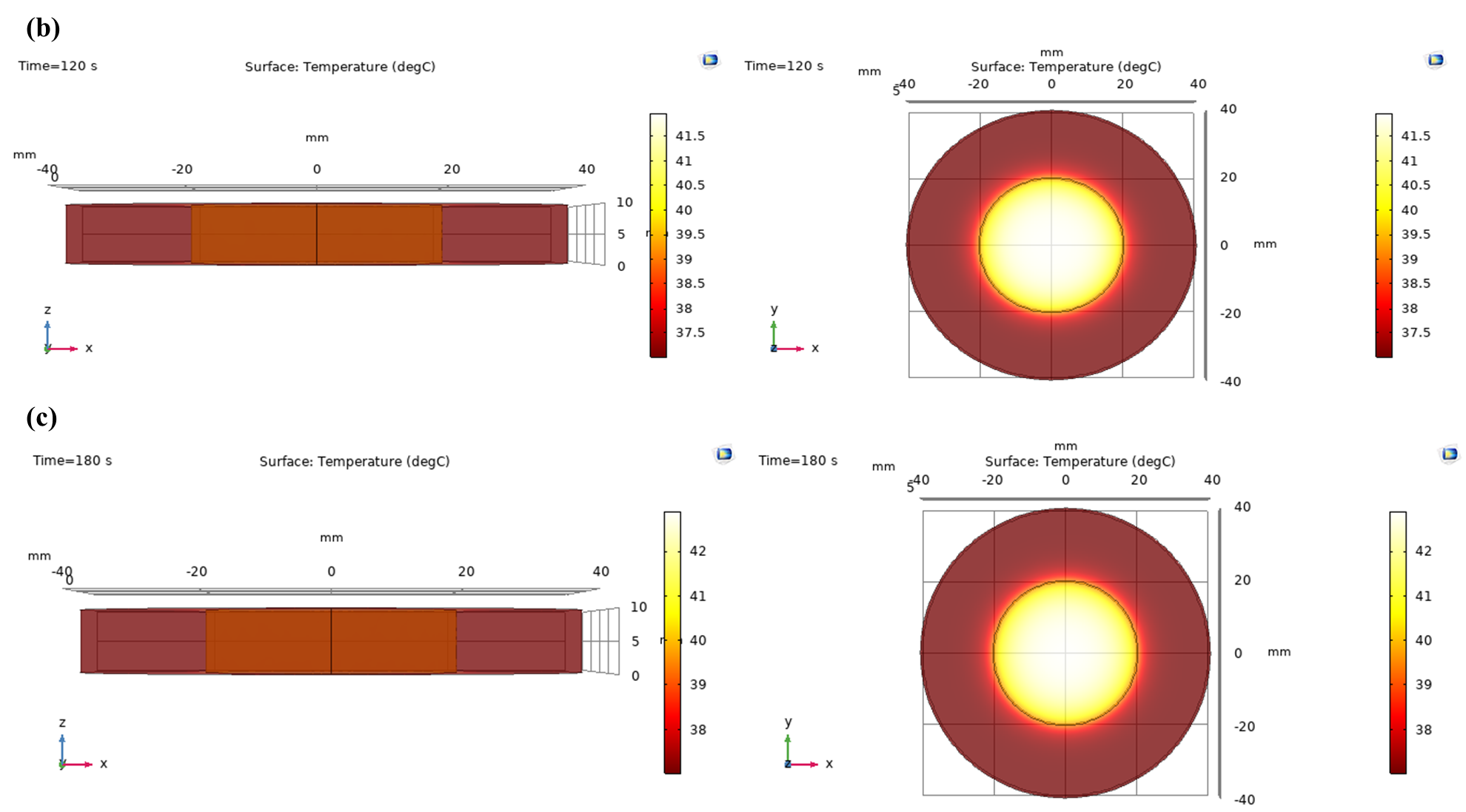


**Figure S3.** Surface temperature plot from COMSOL at the surface of lung tumor tissue in xz and xy planes at irradiation time of (**a**) 60 s, (**b**) 120 s, and (**c**) 180 s by varying the incident flux.


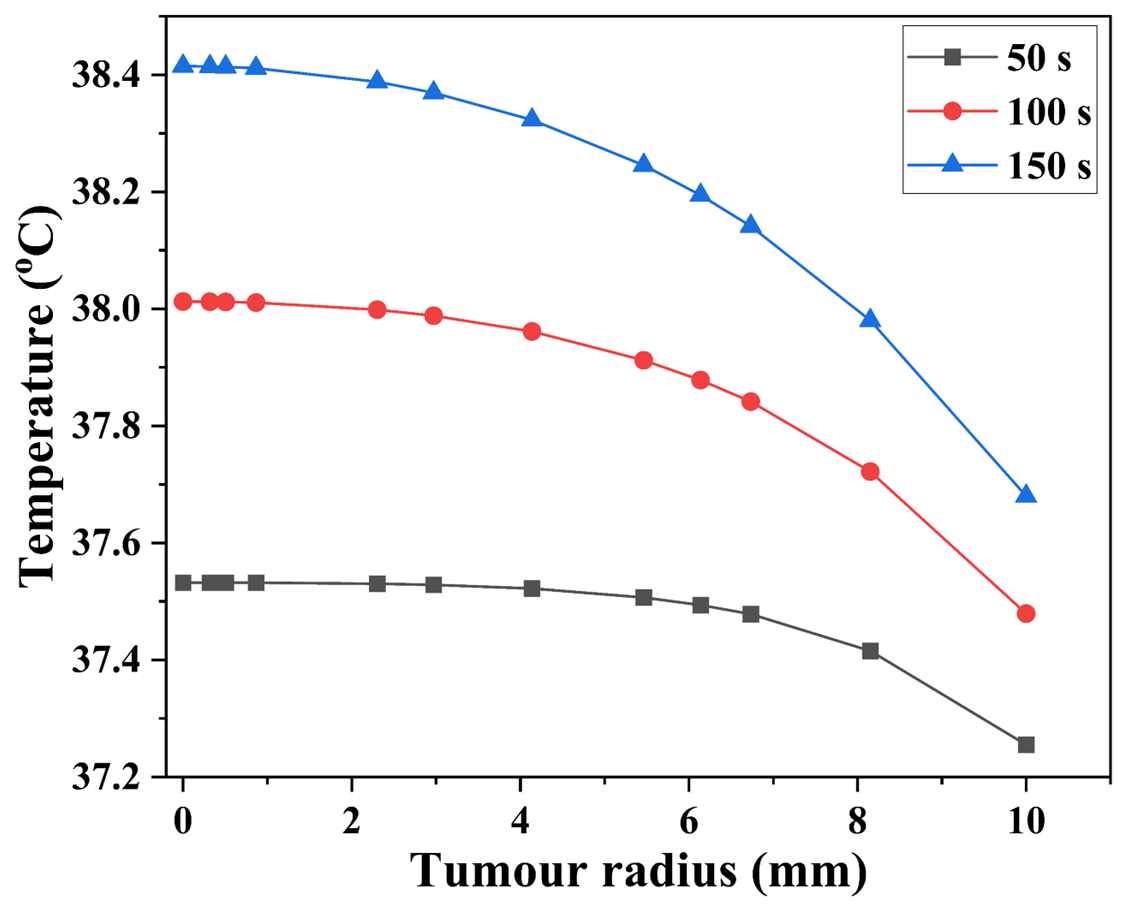


**Figure S4.** Radial variation of temperature at tumor depth **(***Z*) of 5 mm for different time.


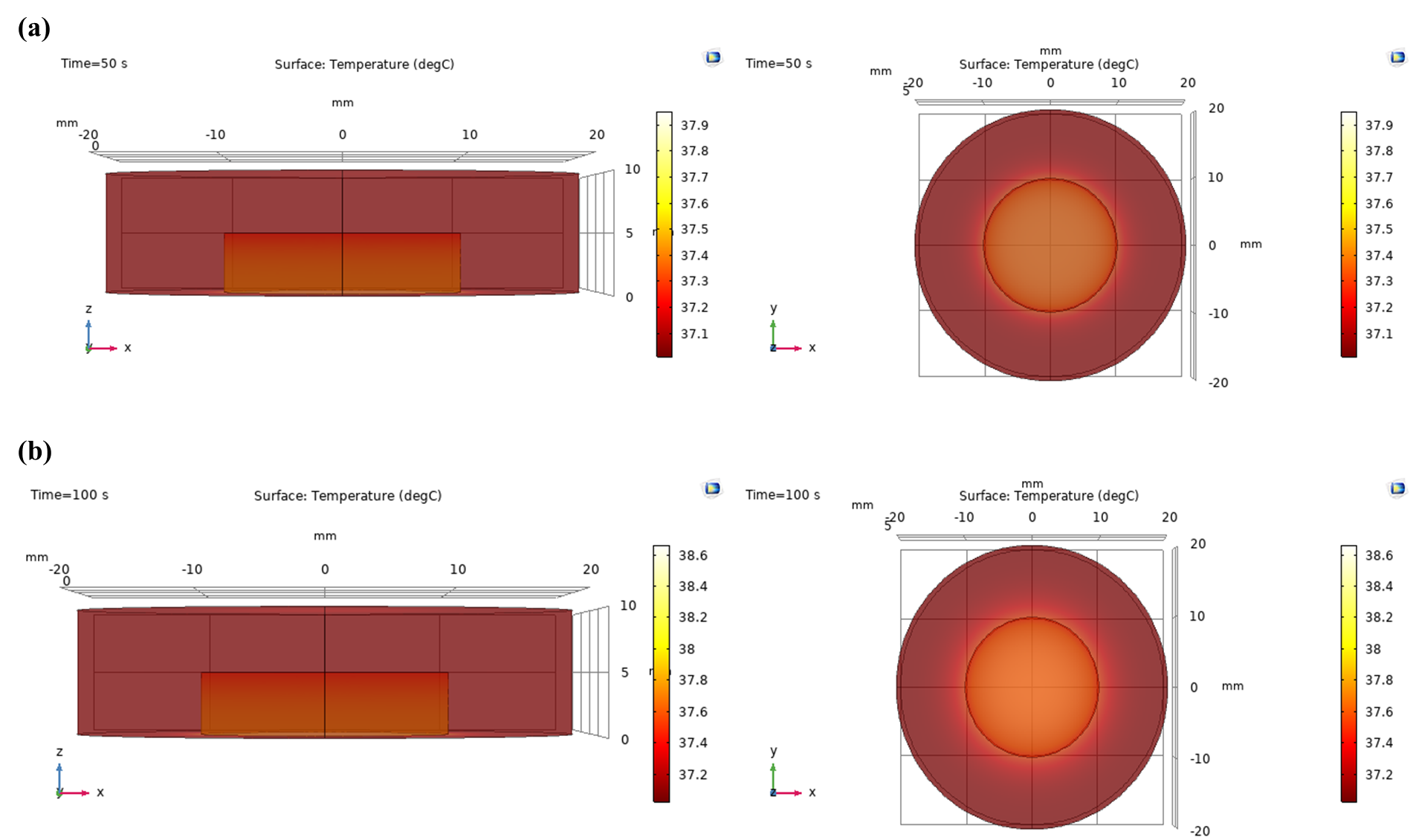

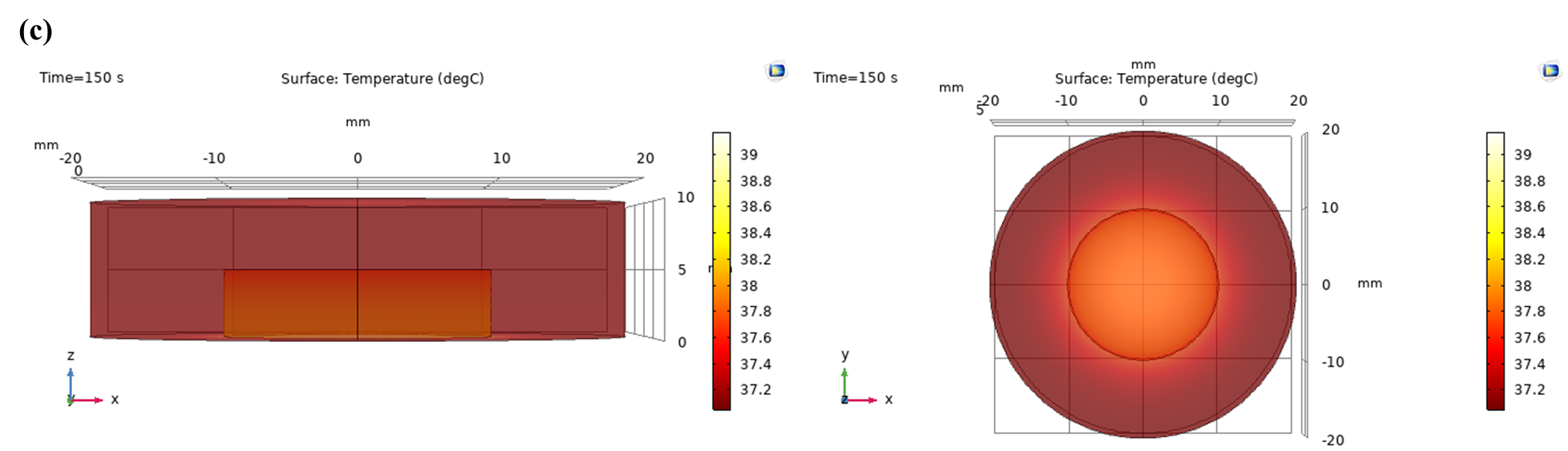


**Figure S5.** Surface temperature plot from COMSOL at the surface of tumor tissue in xz and xy planes at irradiation time of (**a**) 50 s, (**b**) 100 s, and (**c**) 150 s by varying the time.


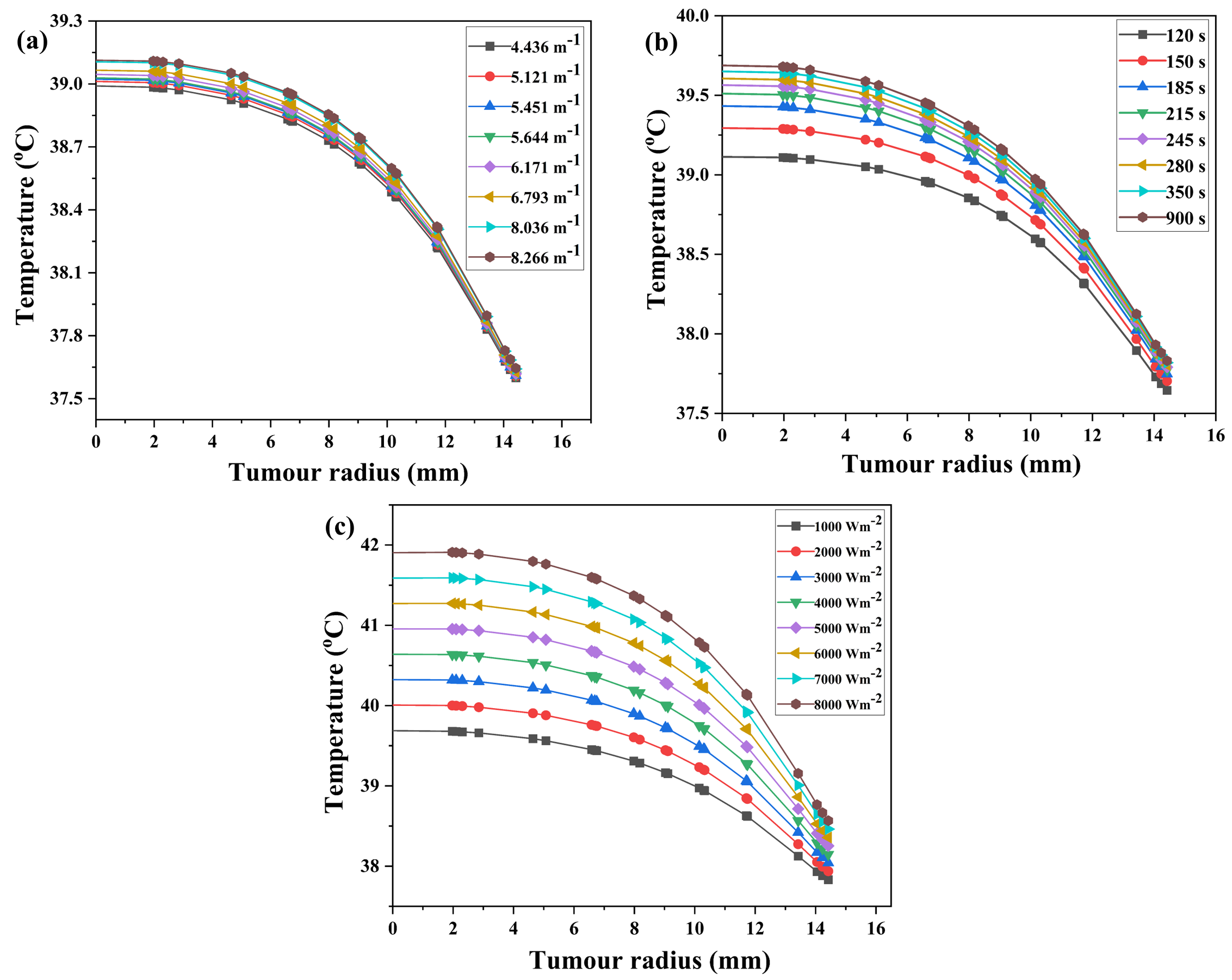


**Figure S6.** Variation in temperature (^o^C) at the bottom (*Z*) of 5 mm of lung tumorous tissue. By varying (**a**) extinction coefficient, (**b**) time, and (**c**) incident flux.


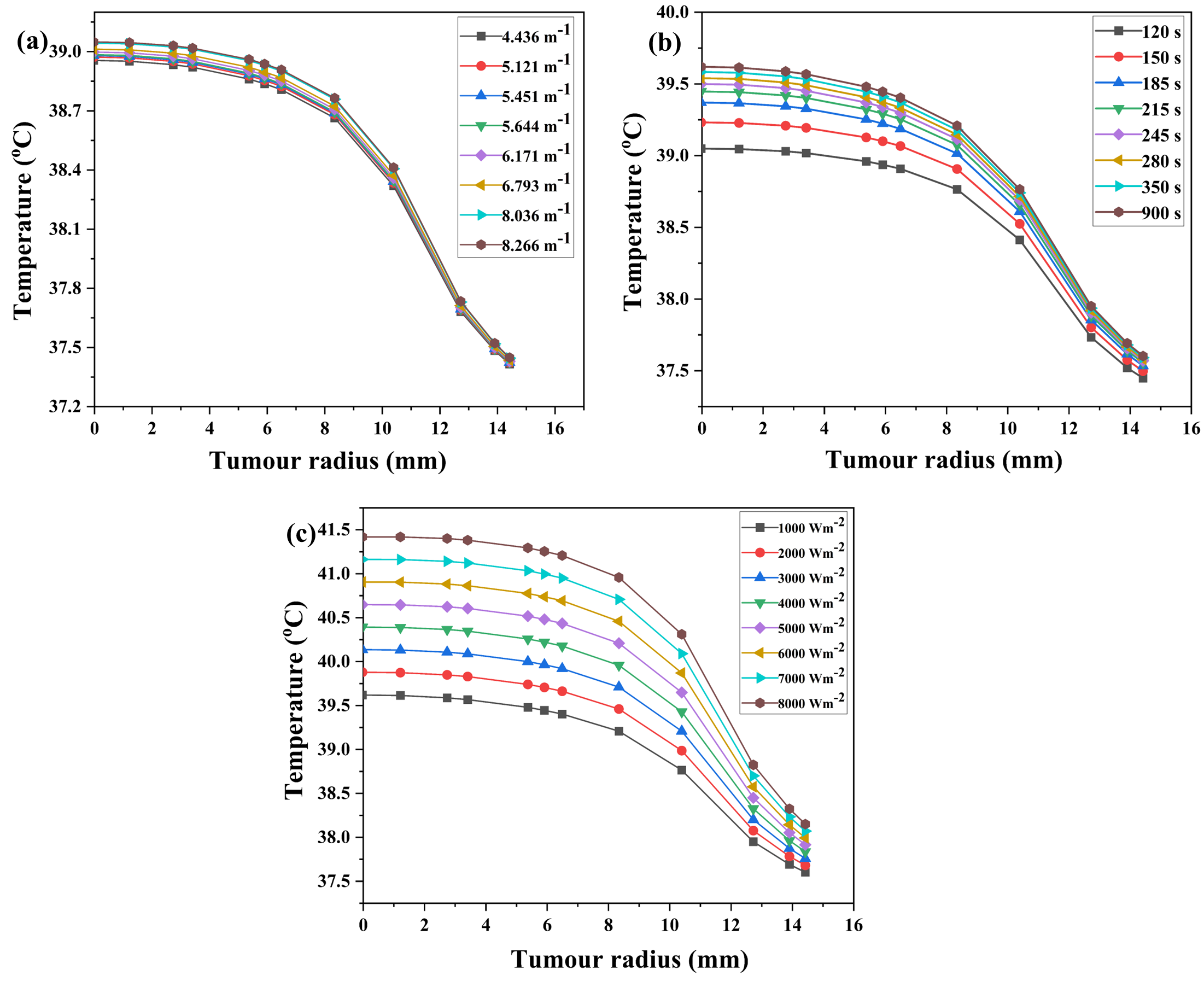


**Figure S7.** Variation in temperature (^o^C) at the bottom (*Z*) of 10 mm of lung tumorous tissue. By varying (**a**) extinction coefficient, (**b**) time, and (**c**) incident flux.

**Table S1.** Characteristic thermophysical parameters of tumor and healthy lung tissue [1,2]

| Property | Tumor | Healthy tissue |
| --- | --- | --- |
| Thermal conductivity (*k*_t_) | 0.39 W/mK | 0.25 W/mK |
| Density (*ρ*) | 385 kg/m^3^ | 241.80 kg/m^3^ |
| Specific heat capacitance (*C*_t_) | 3886 J/kg K | 3800 J/kg K |
| Blood perfusion (*w*_b_) | 3.60 * 10^-3^ s^-1^ | 8.30 * 10^-3^ s^-1^ |

**Table S2.** Symbols and values of other characteristic parameters considered in this study

| Parameter | Value |
| --- | --- |
| Specific heat, blood (*C*_b_) | 4200 J/kg K |
| Density, blood (*ρ*_b_) | 1000 kg/m^3^ |
| Metabolic heat source of tumor cell (*Q*_m_)  Metabolic heat source of healthy tissue (*Q*_m_) | 42000 W/m^3^   1. m^3^ |

**REFERENCES**

1. Liu, J. *et al.* Recent research advances on simulation modeling of temperature distribution in microwave ablation of lung tumors, *Comput. Assist. Surg.* **28**, 2195078. <https://doi.org/10.1080/24699322.2023.2195078> (2023).
2. Giering, K., Minet, O., Lamprecht, I. & Müller, G. Review of thermal properties of biological tissues, Bellingham, *WA: SPIE Opt. Eng.* 45-65 (1995).
